# A comprehensive review of unmanned aerial vehicle-based approaches to support photovoltaic plant diagnosis

**DOI:** 10.1016/j.heliyon.2024.e23983

**Published:** 2024-01-03

**Authors:** Anna Michail, Andreas Livera, Georgios Tziolis, Juan Luis Carús Candás, Alberto Fernandez, Elena Antuña Yudego, Diego Fernández Martínez, Angelos Antonopoulos, Achilleas Tripolitsiotis, Panagiotis Partsinevelos, Eftichis Koutroulis, George E. Georghiou

**Affiliations:** a PV Technology Laboratory, FOSS Research Centre for Sustainable Energy, University of Cyprus, Nicosia, Cyprus; b TSK Electrónica y Electricidad S.A., Gijón, Spain; c Technical University of Crete, Chania, Crete, Greece

**Keywords:** Fault diagnosis, Image analysis, Photovoltaic systems, Unmanned aerial vehicles

## Abstract

Accurate photovoltaic (PV) diagnosis is of paramount importance for reducing investment risk and increasing the bankability of the PV technology. The application of fault diagnostic solutions and troubleshooting on operating PV power plants is vital for ensuring optimal energy harvesting, increased power generation production and optimised field operation and maintenance (O&M) activities. This study aims to give an overview of the existing approaches for PV plant diagnosis, focusing on unmanned aerial vehicle (UAV)-based approaches, that can support PV plant diagnostics using imaging techniques and data-driven analytics. This review paper initially outlines the different degradation mechanisms, failure modes and patterns that PV systems are subjected and then reports the main diagnostic techniques. Furthermore, the essential equipment and sensor's requirements for diagnosing failures in monitored PV systems using UAV-based approaches are provided. Moreover, the study summarizes the operating conditions and the various failure types that can be detected by such diagnostic approaches. Finally, it provides recommendations and insights on how to develop a fully functional UAV-based diagnostic tool, capable of detecting and classifying accurately failure modes in PV systems, while also locating the exact position of faulty modules.

## 1 Introduction

Photovoltaics (PV), that convert sunlight to electricity, will play a dominant role in electricity generation, as it is the fastest growing form of renewable energy source (RES), experiencing significant growth with no signs of slowing down [1]. Currently, the world has reached the Terawatt era for solar energy [1], recognizing the enormous potential of the sun for independent energy supply, climate protection and alleviation of pressure on energy prices. The multi-terawatt PV deployment is thus possible with determined solar energy policies and solar module manufacturing ambitions. To this end and following the PV capacity growth rate, there has been a rapid expansion in the solar technology with new modules featuring different advanced PV cell designs and innovations, that improve efficiency and reliability and reduce degradation over the last few years [2,3]. However, existing fielded PV systems may suffer from different degradation modes and failures (such as open-circuit faults, cracks, shading effects, soiling, dust, etc.) [4]. Such fault modes lower the produced PV power and the lifetime of the installations [5]. As a result, solar modules diagnosis and maintenance is of utmost importance to reduce energy losses, particularly in the context of large-scale PV systems. Accurate detection and classification of such faults at early stage is thus vital for improved energy yield and reduced PV operation and maintenance (O&M) costs through optimised planning of field activities [6,7].

Several PV diagnostic strategies have been proposed in the literature [6,[8], [9], [10], [11], [12], [13], [14]], which are mainly divided into three categories: (a) visual inspection, (b) electrical data analysis, and (c) imaging techniques [6]. Visual inspection is a simplistic method utilised to spot colour changes and to detect visible PV module defects [6]. Delamination, discoloration, bending, glass breakage and soiling (e.g., dust, dirt and bird droppings) can be detected by visually inspecting PV modules [6]. However, other degradation modes and failures (such as potential induced degradation and hotspots) are often undetectable by the naked eye (or visual cameras/sensors employed in the inspection process). More sophisticated diagnostic tools are thus required to detect such performance losses [6].

Electrical data analysis techniques are extensively employed for PV fault detection and classification [[15], [16], [17], [18]]. Such methods operate on the monitored weather parameters, electrical/PV performance data and signals and decide upon fault conditions using statistical and/or comparative analysis methods [6]. Common techniques for fault diagnosis involve outlier detection rules and comparative assessment between the PV system's simulated (predicted) and measured parameters. Data-driven analysis using machine learning (ML) techniques is gaining popularity in the field of PV plant diagnosis due to the availability of sophisticated technologies, networking and sensors (i.e., Industry 4.0 era and internet of things [IoT] sensors) and current innovations in the field of artificial intelligence (AI) [6]. The utilisation of electrical data analysis methods enables the identification of numerous failures, including inverter shutdowns and string disconnections, line-to-line failures, open- and short-circuit faults, performance degradation, shorted bypass diodes, soiling, partial shading, etc. [6]. Advantages of the electrical data analytic methods include the utilisation of existing equipment, automated detection of fault modes, high fault prediction rate (reaching up to 100 % in some cases [19]) and optimization of O&M activities [6]. Despite their ability to monitor online a PV plant and detect faults in real-time, data-driven methods require a training process and they may exhibit large margins of errors (this depends on the simulation model used for predicting the PV performance), causing false alarms [20]. In addition, the accuracy of data-driven diagnostic approaches relies heavily on the availability and quality of the recorded data. Therefore, the data should be pre-processed before commencing the fault detection phase [18]. In addition, suitable selection of the learning algorithm should be performed based on the problem to be detected and desired output (e.g., detection and/or classification of underperformance events, categorisation of failures, etc.) [21].

Finally, the most common failure diagnosis method is based on imaging analysis techniques. Imaging analysis techniques involve the extraction of valuable information and failure results by analysing and processing images. Such images include Red, Green, Blue (RGB), infrared thermography (IRT), ultraviolet (UV) fluorescence, photoluminescence (PL) and electroluminescence (EL). Imaging processing techniques can be used for inspecting the health-state condition of PV modules, while also detecting several failures in PV power plants. IRT and EL imaging are well established methods used in PV applications (e.g., plant's diagnostics), since they can reveal the exact location of the fault by providing high-resolution images of PV modules [10]. Defects and failures that can be detected by both methods include potential induced degradation (PID) and inactive cells. Hotspots are also identified from IRT images, while cracks can be detected from EL images [22]. A combination of IRT and EL image techniques could enable the quick and accurate detection of the most common root causes of defects within a PV module [10].

The inspection methods (visual or imaging) used for PV plant diagnosis can also be categorised into traditional and more advanced automated methods. The traditional inspection method involves maintenance personnel patrolling the entire PV plant for observing visual defects and/or capturing IR/EL images (using hand-held cameras or tripod system) for the detection of malfunctioning PV modules [10,23]. The O&M annually costs of a traditional inspection are estimated to be around 11.30 €/kW_p_ [24], from which only 1.50 €/kW_p_ are the costs associated to the planned monitoring and inspection. Bearing this in mind, more advanced and automated methods and tools are becoming increasingly popular lately. In this domain, unmanned aerial vehicles (UAV), fitted with cameras, sensors and control systems, are employed to inspect and monitor large areas with fielded PV systems. Monitoring of installed PV arrays by UAV offers many advantages, including fast detection, precise imagery, low cost and large area coverage, unmanned operations, high flexibility, ability to operate in harsh environments and unreachable areas/locations [23]. In addition, a noticeable time reduction is achieved when monitoring PV arrays using UAV. To this end, a large-scale PV system can be inspected for defects in a shorter time.

Over the past decades, advances in the aerial industry technology along with the improvements in control systems and sensor technology, have led to the substantial usage of UAVs (equipped with thermal and/or EL sensors/cameras) for PV monitoring and O&M applications. As such, the UAV system is increasingly becoming the accepted norm for gathering data and inspecting PV systems for common faults [25]. The use of a UAV diagnostic system can increase the inspection efficiency by up to 97 % on average compared to the manual inspection [26] (i.e., 1.10 €/kW_p_ reduction in the O&M annually costs [27]). Also, it can ensure optimal lifetime PV performance by monitoring the health-state of PV components in real-time and detecting failures at early stages, which is crucial for O&M cost (and LCOE) reduction (through the optimization of field O&M activities). The reduction of produced energy due to malfunctions, failures and/or performance losses can drastically reduce the expected revenues and the overall reliability of the PV installation [2,28,29]. For instance, a 270 GW_p_ plant with 4 % loss in energy yield can cause economic losses up to 2 billion $/year [30]. Therefore, early fault diagnosis (detection and classification) using a UAV inspection system is crucial for PV plant's O&M to ensure adequate performance, prevent extension of defects to healthy areas and reduce the monitoring cost. Though, suitable identification of the UAV characteristics is required for reliable, time- and cost-effective inspection of PV plants.

A literature search revealed several diagnostic strategies for grid-connected PV systems [16,22,[31], [32], [33], [34], [35], [36], [37], [38]]. The published research mainly dealt with the different methods (electrical data analysis, visual inspection and thermal images) for diagnosing failures in PV systems. Nowadays, researchers are working to explore more advanced ML algorithms (e.g., deep learning, gradient boosting, etc.) and image analysis techniques to tackle the challenge of complete and automated fault diagnosis for large-scale PV plants. Related review papers and reports [9,10,13,[39], [40], [41]] published in the field either focused on only one specific method (e.g., imaging techniques [13,39,40], electrical characterization methods [9,10,41]) or failed to present an extensive review of the latest UAV-based approaches, that can combine both imaging techniques and electrical data analytic methods for PV plant diagnosis. Also, none of the published review papers in the literature focused on the identification of the appropriate UAV characteristics for PV plant inspection and field applications. This review paper thus contributes towards the identification of useful information for fully functional UAV systems, capable of diagnosing accurately failure modes in PV systems, bridging the knowledge gap in the field of UAV-based diagnosis and inspection. Additionally, this work outlines the state-of-the-art image techniques (along with their effectiveness in detecting faults) and UAV-based systems for PV plant diagnosis. In this light, the suitable identification of the UAV characteristics is also provided for reliable inspection and cost-effective fault diagnosis in PV power plants.

The methodology followed for this review paper consists of four steps. In the first step, a keyword search was performed. Keywords used included UAV, PV plant inspection, fault diagnosis, image techniques, infrared thermography, EL and RGB. As a second step, screening of the papers obtained from the first step was conducted to ensure that the studies were related to UAV-based systems that can be used to support PV plant diagnosis. The third step was to review all the selected articles, to reveal their objectives and to summarise the techniques used for PV plant diagnostics. The final step was to summarise the findings reported in the literature and to identify the optimal methodologies for UAV-based approaches for PV plants’ inspection and diagnosis.

The rest of this paper is structured as follows: Section 2 describes the main fault modes along with root causes. It also provides an overview of the existing approaches for fault diagnosis in PV systems. Section 3 focuses on the main image processing techniques and UAV platforms reported in literature for PV plant diagnosis. An overview of different PV diagnostic approaches based on image processing techniques are summarised in Section 4. Results and discussion of the finding of the present study are given in Section 5. Finally, Section 6 concludes the paper and provides recommendations and insights for the development of functional UAV-based tools for diagnosing faults in PV systems.

## 2 Failure modes in PV systems and existing approaches

### 2.1 Failure modes in PV systems

**Fig. 1:**
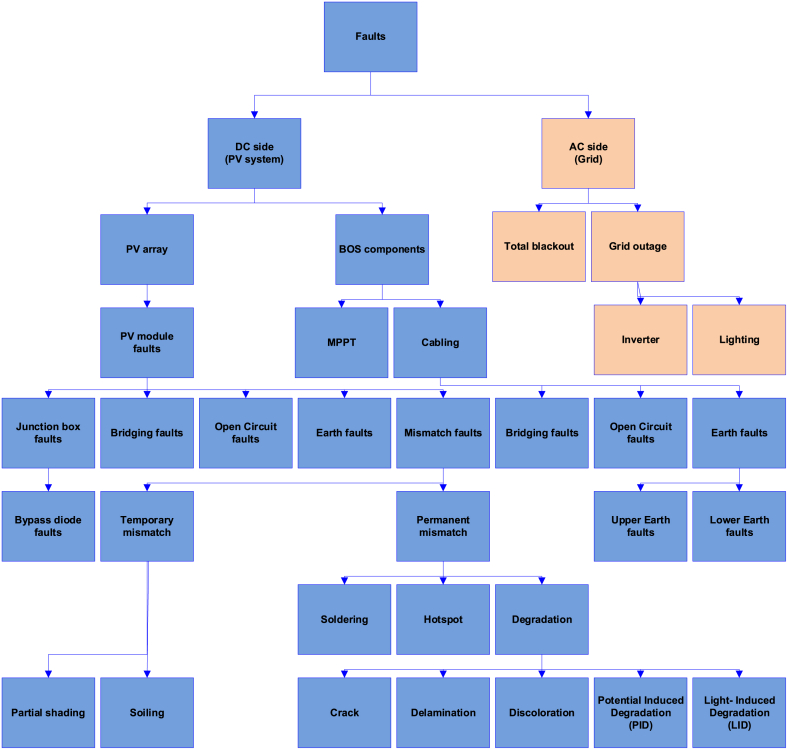
Failures categorisation in grid-connected PV systems based on their structure and location [6].

Various failure modes can occur during the operation of a PV system. A failure mode (also known as a fault) is characterized as an occurrence that reduces the power output of a PV system, contributing to power losses and/or causing safety problems [42]. Failures can occur at both sides (AC or DC) of the system. Failures occurring at the DC side can be further classified into PV array and module/cell level failures, depending on the location of occurrence (e.g., a crack and a hotspot occur at the cell(s) level) [43]. According to Livera et al. [6], failures classification is mostly based on their structure and location (see Fig. 1).

**Fig. 2:**
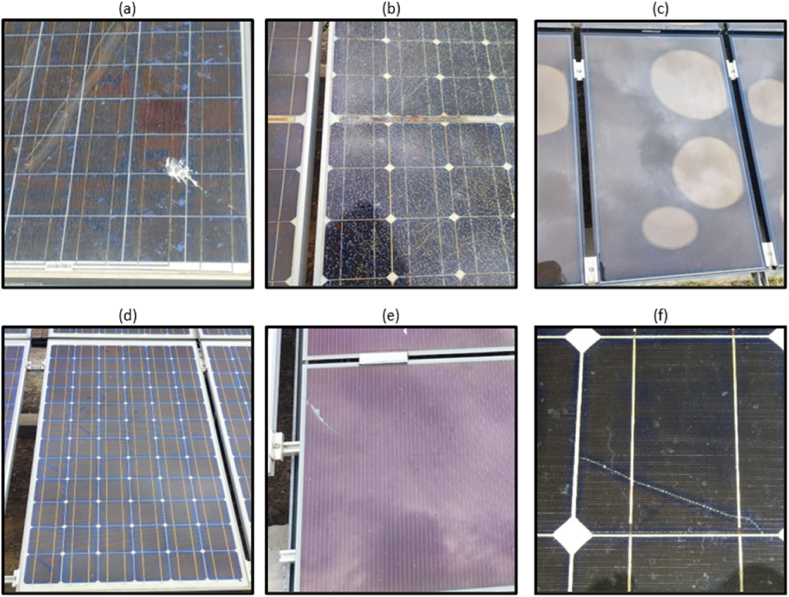
Visible PV module related failures: (a) bird dropping (soiling), (b) glass breakage, delamination and corrosion, (c) discoloration, (d) browed EVA, (e) glass breakage, and (f) cell crack at PV cell (snail trail). The images were taken at the PV Lab of the University of Cyprus.

Faults can manifest as performance deficiencies, safety hazards, or faults associated with PV modules [44]. Common failure modes affecting the PV modules include bird dropping and soiling (Fig. 2a), delamination and corrosion (Fig. 2b), discoloration (Fig. 2c), browning of the ethylene vinyl acetate (EVA) as shown in Fig. 2d, glass breakage (Fig. 2e), hotspots, snail trail and cracked cells (Fig. 2f). The phenomenon of delamination depends on the separation of two layers of the panel, the polymer glass set and the back sheet [45]. Delamination occurs frequently due to the entry of humidity into the PV module, causing a local reduction in thermal conductivity, thereby increasing cell's temperature. Moisture ingress in the PV modules due to the delamination leads to the occurrence of different sort of physical and chemical degradation (e.g., corrosion in the metallic section) [44].

The discoloration of the polymer or EVA layer is due to its exposure to UV radiation at high temperatures (i.e., 85–90 °C) [46]. With discoloration of the polymer, the short-circuit current (I_sc_) decreases due to the lower light transmittance. The presence of discoloured areas also reduces the amount of total current that can flow through the cell. Due to the serial connection of cells and despite that the discoloration is present in only one of them, this condition will be generalized to the current of the entire string [47]. Numerous studies have concluded that discoloration by itself does not affect the performance of solar modules; the decrease in performance is generally related to micro-cracks, since these can end up affecting the components of cells [42,48].

Cells cracks are also a potential occurrence throughout the lifespan of a PV module [49]. Manufacturing is a main reason for cell cracking (e.g., the soldering process can result to excessive pressure on PV cells). Another cause for the creation/expansion of cracks in PV modules is the handling and vibration during the transportation from the factory. In addition, extreme weather conditions (e.g., snow loads and heavy wind) can lead to mechanical loads on fielded PV modules due to the posed vibration and pressure, resulting in cracks on PV modules surface.

The primary cause of glass breakage in PV modules often relates to the clamps used in the installation process. The breakage of glass can lead to an electrical security problem, and/or to the creation of hotspots [42]. Another failure mode affecting PV modules is the snail trails, which are discoloured lines that occur in the surface of PV modules. Snail trails are usually caused by a chemical process that causes oxidation and small dark lines. Environmental conditions and errors in the manufacturing process are main causes for the appearance of snail trails. The snail trail is commonly associated with micro-cracks, since these discoloration marks tend to draw a line that runs even to that of micro-cracks. Though, it is frequently manifested at the edges of the PV cell. It is generally accepted that the appearance of snail trails signals the existence of micro-cracks but not necessarily the reverse [50].

PID occurs when there is a significant potential difference between the frame of a solar module and its array. This phenomenon is more frequently observed in hot and humid climates, since the combination of voltage, heat and humidity usually gives rise to this problem after several years of field operation [42,51]. The voltage difference generates a leakage current of ions that generally occurs from the glass layer and runs through the EVA encapsulating layer and anti-reflective coating. The current affects the solar cells, causing a degradation in the production capacity of the panel that can be quite significant (e.g., up to 30 %) [42]. At the module level, the degradation usually evolves from the solar cells closest to the aluminium frame (where the effect is greatest) and then spreads to the central cells, which are generally less affected [52].

Bypass diode failures is another common problem in PV systems. Bypass diodes, located in a junction box (JB), protect the cells in a string from hotspots and/or shadowing. They are used to maintain the power flow in the intended direction and to block it from feeding back to the modules in absence of light in order to avoid underperformance effects caused by mismatches, hotspots, and shading [53].

Welding defects in the joints are another failure mode produced by a “bad/poor” welding process and they can cause conductivity problems for the components, leading to hotspots. Additionally, in PV plants there are some common electrical failures that can occur, including ground faults, line-line and mismatch (or open-circuit) faults [54]. Furthermore, as the performance of PV modules is affected by the received irradiation, soiling, snow deposition and/or shading can clearly affect the PV production. Soiling refers to the accumulation of dirt, dust, pollen and/or other elements on the PV modules surface. It is a major performance loss type, especially in areas that exhibit heavy dust deposits or sandstorms [55]. According to the International Energy Agency (IEA) [56], after irradiance, soiling is the most influential parameter impacting solar PV system yield. It is estimated to cause up to 5 % decrease of the annual PV energy yield. This corresponds to an economic loss ranging from 3 to 5 billion €/year. Regarding snow deposition, issues arising include total/partial obstruction of irradiation, cracking and delamination or mechanical loads.

Lastly, hotspots are defined as areas of the cell that present a temperature significantly higher than that of their environment, potentially causing damage to the cell and system elements [57]. The most typical causes include cell misconnection and partial shading. In the first case, it is assumed that all the panel cells connected in series have identical characteristics and operate on the same current (that of the maximum power point). When, one or more cells generate current less intense than the rest, there is a decompensation in the series causing higher voltages and hence higher cell temperature. Consequently, a hotspot is produced, that is an overloaded and overheated cell area with lower current. The most frequent consequences of this phenomenon are usually limited to a localized reduction in the efficiency of the affected modules and a greater degradation of the materials that would shorten their useful life. On the other hand, when cells are subjected to partial shading (due to nearby arrays, trees and vegetation, buildings and other light-blocking obstacles), they behave as charges rather than generators, as they can experience reverse currents. These hotspots can also occur due to “shunt resistance”, which appears due to a short-circuit or an error in the connections during the manufacturing process. Hotspots can also be due to structural defects in the manufacture of the panels (poorly welded connections, delamination, material defects, etc.) or mechanical damage as a result of improper handling or transportation (micro-cracks, metal frames bent, etc.) [50].

The detected failure modes affect the PV output power production. Depending on the affected electrical signal, the detected underperformance incidents can also be categorised into three main categories; current, voltage, and/or power (that affect both the current and voltage) related failures [4]. Current related failures include homogeneous or heterogeneous loss of transparency, glass corrosion, delamination, coating corrosion of the cells and cracked cells [42]. On the other hand, voltage related failures include inverted or short-circuited bypass diode, short-circuited cells, passivation degradation, PID and LID [42]. Finally, power related failures include solder corrosion, broken cell interconnect ribbons and homogeneous soldering disconnections [42].

### 2.2 Approaches for failure diagnosis in PV systems

Although significant advancements were made in the PV module technology, the performance of PV modules under field exposure has raised concerns [50,58]. Moreover, predictions indicate that approximately 2 % of the PV panels will fail to meet the manufacturer's warranty after operating for 11–12 years [53]. Consequently, it is vital to develop tools for identifying the fault root causes during the PV operation and to isolate and treat the occurred incidents by executing mitigation activities (e.g., corrective/preventive actions) [59]. The early and fast diagnosis of PV faults has become a technical and economic challenge, with the PV assets continuing to underperform by up to 8 % [60,61].

The reported PV fault detection and diagnosis (FDD) methods can be categorised into three main categories: visual inspection, image techniques and analysis of the recorded electrical/PV performance data. More details about each category, the fault modes that can be detected by each method and the reported accuracies are given in the following subsections.

#### 2.2.1 Visual inspection

**Fig. 3:**
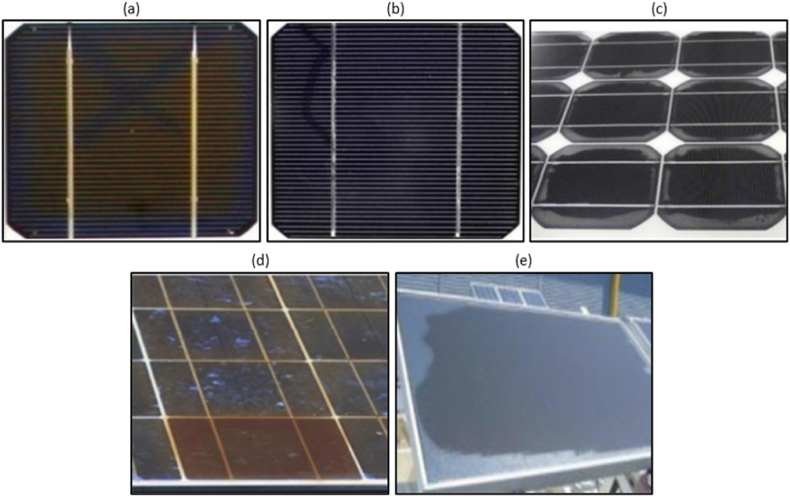
Visible types of defects in PV modules (a) browned EVA with two cracks, (b) snail trails, (c) delamination, (d) browned PV cells, where a single cell is browner when it is hotter compared to other cells, and (e) corrosion. Figure recreated from Ref. [44].

Visual inspection method is utilised to detect visual defects and colour changes, such as browned EVA and cracks (Fig. 3a), glass breakage, snail trails (Fig. 3b), delamination (Fig. 3c), discoloration - browned PV cells (Fig. 3d) and corrosion (Fig. 3e), by naked eye (or by a visual camera). It can also be used to detect temporary failures such as dirt, dust, bird droppings, leaves and shadow. During the inspection process, the whole PV installation (e.g., PV modules, frame, tracking system, electrical installation, cables, connection boxes, fuses, surge arresters, etc.) is inspected for defects and frequent visual checks are required. It is worth noting that many underperformance incidents and degradation mechanisms (e.g., PID and hotspots) are often invisible to the naked eye.

#### 2.2.2 Image techniques

Image techniques include the analysis of thermal, RGB, and EL images to derive useful information for the object under study (e.g., PV installation). Suitable equipment is needed for acquiring images of the PV installation under inspection and nearly all known PV system/module failures can be detected. Such methods can also determine the fault root cause, while also indicating the precise location of the faulty PV module with high accuracy [62]. With the current trends and innovations in the O&M sector, the cameras are attached on drones (remotely piloted aircrafts [RPAs] or UAV) to capture such images [63]. In the last few years, such aerial monitoring techniques are gaining popularity for inspecting PV plants and diagnosing the health status of PV components, thus revolutionise the PV O&M operations [63].

IRT imaging involves gauging the infrared radiation (IR) discharged from a body's surface [41]. Thermal imaging cameras are used to produce an image (and/or a video), displayed as a photograph of the temperature of the radiation. The thermal cameras measure the PV module surface temperature and produce an image (called a thermography or thermogram) with colours, that can be easily interpreted, showing thermal patterns and behaviour. IRT is a very effective inspection tool since the defected PV modules usually experience deviation in their temperature distribution [64]. Thermal imaging is usually performed during daylight hours with minimum radiation levels [44]. Though, this depends on the manufacturer's specifications.

Thermography can be conducted using the active or the passive approach [65]. The technique of active IRT utilizes an external source to increase the temperature of the object under study. The primary types of active IRT encompass pulsed, lock-in, long-pulse and vibro-thermography. The most common one is pulsed IRT due to the easy and quick application. In this approach, the body is heated by a heat pulse, produced with lamps, heating gun, flashes, etc. Then, the temperature data are acquired during the cooling process (i.e., when temperature decreases) of the object. In lock-in IRT, the heating of the object is achieved using an oscillating temperature field. An internal failure is detected by this technique when the received waves vary. However, this method also necessitates coordination between the initial input (thermal source) and the resulting output signal (thermographic signal). Long-pulse (or step heating) IRT uses a continuous low power heat source. Long-pulse differs from the pulsed IRT on the evaluation stage of the object. Specifically, long-pulse focuses on the cooling process, whereas pulse IRT assesses the heating process. Lastly, vibro-thermography uses mechanical vibrations to produce hotspots in defected areas (e.g., cracks, voids, etc.). On the contrary, passive IRT uses the IR emitted by the object, without the need for an external heat source. Passive IRT is a commonly used technique for PV plant diagnosis, especially for large-scale installations [66], due to the easier and faster diagnostic approach compared to the active IRT. Though, active IRT can detect more failure types (e.g., little hotspot, leakages in silicon wafers produced by the fabrication process) than passive IRT.

**Fig. 4:**
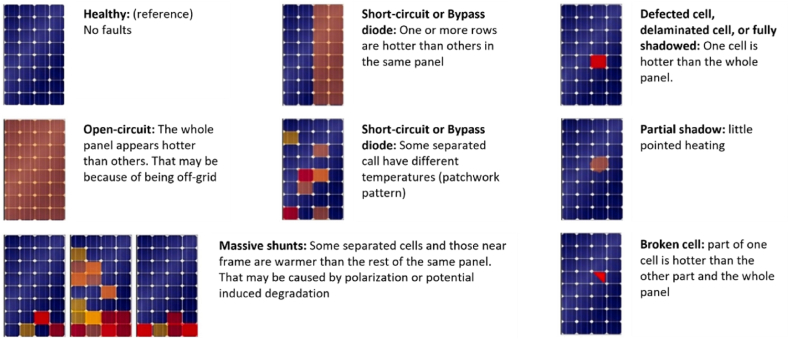
Type of defects and thermal patterns. Figure recreated from Ref. [65].

IRT images for PV applications, provide information about the temperature increase in affected areas and indicate their location on the panel. Most of the faults that occur have a substantial effect on the thermal behaviour of the PV panel. Therefore, faults are identified as inhomogeneities of its surface temperature distribution, visualised in the thermal image of the faulty module [41]. Common PV failures can be identified by knowing the thermal patterns of the failures. Fig. 4 presents some of the thermal patterns for common fault types (such as open- and short-circuit fault, bypass diode failures, partial shading, etc.). Thermal patterns were identified and classified in previous works (e.g., Ref. [67]) based on structural levels as array, module or cell level [43]. More details about the failure patterns are also given in the International Electrotechnical Commission (IEC) TS 62446-3 standard [68].

**Fig. 5:**
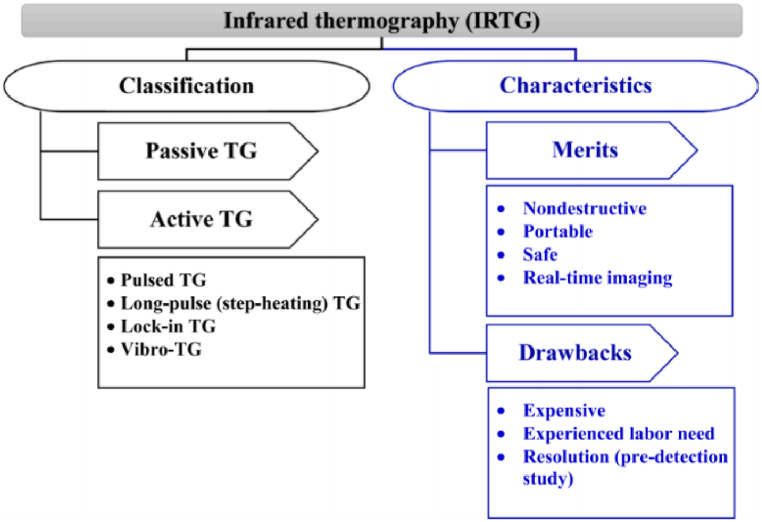
Classification and characteristics (merits and drawbacks) of IRT [66].

Overall, the technique of IRT provides the ability to inspect a PV plant and to accurately diagnose several fault root causes even during daylight hours. Fault modes that can be detected include open-circuited modules, internal short-circuits, bypass diode problems, PID, partial shading, delamination, broken cells, invisible cracks and hotspots [6]. Implementing this technique does not disturb the normal PV plant operation. It is considered to be a safe method for both the personnel and the PV plant under inspection [66]. Additionally, the thermal cameras are portable, meaning real-time images can be obtained. Even though IRT is undeniable a very promising and effective technique for fault detection, its application requires additional equipment (e.g., thermal camera). Besides, well-trained personnel are also needed to handle the cameras and set accurately the required parameters’ settings. Additionally, conducting a preliminary study to determine the relationship between altitude and image resolution for early detection purposes is required [66]. Fig. 5 illustrates an overview of the different existing IRT approaches as well as the merits and drawbacks of this technique. Recent studies [23,27,67,[69], [70], [71]] have shown that thermal cameras embedded in UAVs along with the usage of more advanced image analysis techniques can overcome the needs for expert personnel, while also automatising the PV fault diagnosis procedure. More details for such procedures are given in subsection 3.1.1.

Another imaging technique for fault diagnosis is the analysis of RGB images. This technique is a very similar approach to visual inspection (the same failures can be detected as in visual inspection by naked eye). It processes captured colour images. Automated inspection of PV installations is performed by using a RGB sensor/camera, that can also be embedded to UAV platforms. As reported in the literature [62,[72], [73], [74], [75], [76]], the inspection of RGB images is usually performed along with IRT for aerial plant inspection. Over the past years, researchers and operators have been using RGB cameras embedded to UAV as a standalone fault diagnosis technique [25,[77], [78], [79], [80], [81]].

RGB images can be processed to detect and classify different failure modes, including soiling (i.e., dust, bird droppings etc.), partial shading, burn marks, delamination, discoloration, snail trails, glass breakages, and cracks [78,79]. Furthermore, the use of both RGB and thermal cameras for aerial inspections was proved to be an improved solution for plant diagnosis, offering faster and easier classification of faults compared to the usage of only IRT images [82]. It was also demonstrated that even though the integration of an additional RGB camera increases the cost of a UAV platform, this cost is offset by the reduction of inspection time, and therefore, decreasing workmanship costs [82].

EL involves the application of a direct current (supplied by an external power source) to the PV module and measuring the photoemission by means of an infrared-responsive camera [10]. This procedure is typically carried out in a dark environment due to the low amount of near IR emitted by the PV modules compared to the emitted radiation by background light and sun. Thus, on-site EL imaging must be performed during nighttime or while using a tent to cover the PV modules. A typical structure is composed of a camera, a tripod, a portable DC power supply and extension cables [10]. Moreover, a high pass edge filter may be used for reducing the interfering light from other sources.

**Fig. 6:**
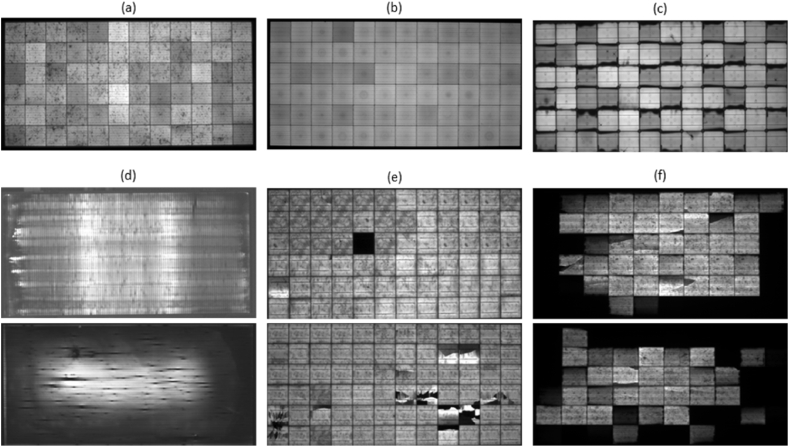
EL images of healthy and defected modules of different PV technologies, (a) healthy multicrystalline silicon (multi-*c*-Si) PV module, (b) healthy monocrystalline silicon (mono-*c*-Si) PV module, (c) mono-*c*-Si PV module with shunted cells, (d) thin film PV modules with degradation of the TCO (halo-like appearance) and edge degradation, (e) multi-*c*-Si PV modules with broken cells, cracks and hotspots, and (f) multi-*c*-Si PV modules with PID. The images were taken at the PV Technology Lab of the University of Cyprus. (For interpretation of the references to color in this figure legend, the reader is referred to the Web version of this article.)

EL images can be processed to assess the health-state of PV modules (EL images of healthy PV modules from indoor testing are shown in Fig. 6a–b) and to detect cracks, micro-cracks and other defects within the cell material (see Fig. 6c–f), which are not discernible to the human eye. The arrangement of brightness in the EL images is correlated with the distribution of the open-circuit voltage, the minority carrier diffusion length, the series resistance, the quantum efficiency and the ideality factor of the tested cell. Fig. 6 depicts EL images (obtained from indoor testing) of healthy and defected (i.e., shunted cells as shown in Fig. 6c, degradation of the transparent conductive oxide (TCO) as shown in Fig. 6d, broken cells, cracks and hotspots as shown in Fig. 6e, PID as shown in Fig. 6f and edge degradation) PV modules of various technologies (e.g., thin film and crystalline silicon).

If there is a crack/defect in a PV cell/region, the flow of the current is reduced or blocked during forward bias condition at that location based on the type and seriousness of the defect. Such region emits reduced or zero near IR due to the reduced (or zero) electron-hole recombination. That defected cell/region is depicted darker in the EL image since it releases less radiation. Oppositely, normal electron-hole recombination occurs for normal operating cells during forward bias, thus emitting relatively more radiation. Those cells are shown brighter in EL images [83].

EL images have been widely used both indoors and outdoors by several research institutions and companies for identifying problematic PV modules [[84], [85], [86], [87], [88], [89]]. Recent advances in this field include outdoor EL imaging or aerial EL inspection, by using EL cameras mounted on drones (e.g., UAV) and without dismantling PV panels. A main concern when collecting images using a UAV platform, is the perspective distortion of the PV panel, which can affect the performed analysis and the accuracy of the failure diagnostic stage. In this context, Mantel et al. [5] suggested a method to automatically rectify the perspective distortion in EL images of solar modules acquired from UAV. The proposed methodology included the identification and correction of the two major situations of perspective distortion, when the imaging plane was parallel to the panel plane or not. This method estimated the panel rotation angles in the original image, relative to the normal perspective in mathematical terms and then applied a perspective transformation. One drawback of this methodology is the assumption that the PV panel under study constituted the main structure of the image. Nonetheless, in outdoor setups, the PV modules are connected to form arrays. To adapt it for PV arrays, the suggested process needs to be preceded by a PV module identification and segmentation step. On a later study [90], Mantel et al. presented two additional methods to derive the distortion due to perspective on EL images of PV modules, using either four panel or cells corners. In terms of performance, those methods performed well as visually inspected. The cell method was more advantageous in sense of further cell-level processing for fault detection. However, both methods performed satisfactory on PV images captured by a drone.

#### 2.2.3 Electrical data characterisation methods

Electrical data analytic methods involve the analysis of system's performance (i.e., electrical) data/signals and weather parameters. Such data are readily available through the monitoring platform and no additional equipment or labour expenses are required. Such methods use electrical signatures for fault detection in PV systems and they can be utilised for online monitoring and diagnostics, even for large-scale PV installations. One of the main challenges is the difficulty to locate the fault root cause [91] and the exact position of the problem/affected module [92].

**Fig. 7:**
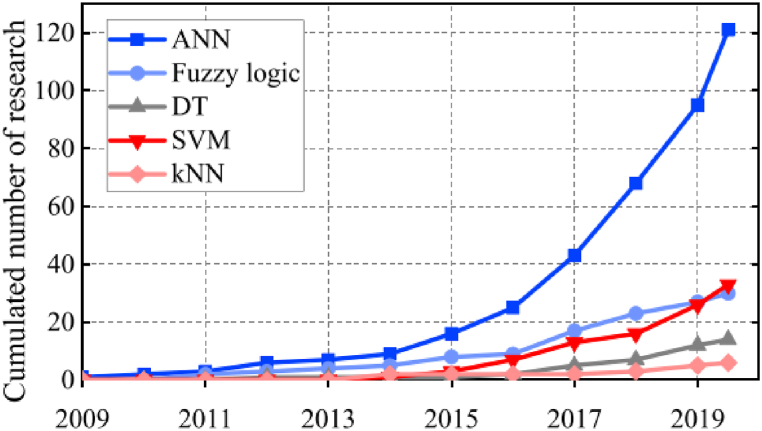
Applications of ML techniques for PV FDD [43].

Electrical data characterisation methods include I–V curve analysis [93,94], signal analysis, circuit and simulation models [6]. Current trends and innovations in today's O&M market include the application of ML techniques [38,43,63,85] and data-driven decision making processes [[95], [96], [97], [98]]. The number of ML-based research over the years for PV FDD is illustrated in Fig. 7, showing the popularity gained in the last few years [43].

In this domain, Li, B. et al. [43] conducted a literature survey on ANN methods for FDD in PV systems. The ANN based methods were efficient for the diagnosis of common PV faults, such as electrical faults and permanent visible faults (cell crack, discoloration, delamination). This study also indicated the main challenge to configure the model because of the limited accessibility to open-source PV data (with and without labelled PV failures).

Similarly, Mellit, A. presented a review of AI techniques for PV fault diagnosis [21]. The main AI techniques were categorised into evolutionary algorithms (e.g., genetic algorithm [GA]), ML, neural networks (e.g., RNN), fuzzy logic, deep learning (DL), and hybrid systems (i.e., two or more AI techniques combined with DL) [21], with the ML and DL techniques being the most preferable. Most of the reported techniques included neural networks and fuzzy logic due to their ability to distinguish different fault types with the same fault pattern.

Gnetchejo, P. J. et al. studied the use of kernel principal component analysis (KPCA) for PV fault diagnosis [20]. The KPCA was applied on recorded and estimated data of a PV array to identify and classify faults in 5 different sources groups. The results showed that the accuracy could vary depending on the fault type and the irradiance used for each test set. Pahwa, K. et al. [99] examined several ML techniques (i.e., extreme gradient boosting [XGBoost], decision tree, random forest and neural networks) for fault detection and classification, with neural networks achieving the optimal accuracy.

Hussain, M. et al. in Ref. [100] presented a successful algorithm for fault detection based on shading environmental conditions with only two parameters as inputs (irradiance and power output) using a radical basis function (RBF) as the base for the neural network. Ul-Haq, A. et al. in Ref. [3] investigated the use of multilayer neural networks with a scaled conjugate gradient (SCG) algorithm to categorize faults on PV arrays. The developed methodology showed high classification accuracy and fast computational time (0.10 s). Basnet, B. et al. [19] constructed a PV fault detection model using probabilistic neural network (PNN) to classify major electrical fault types.

In the same context, Mellit, A. and Kalogirou S [85]. investigated the application of various ML and ensemble learning techniques for fault diagnosis in PV systems. The algorithm was capable of classifying fault occurrences such as partial shading, degradation, soiling, short-circuit, open-circuit, line-to-line [85]. No significance differences were observed between ML and ensemble learning methods, with ANN, advanced stacking method and CatBoost being the best performing models, respectively.

### 2.3 Failures that can be detected by each method and reported accuracies

Table 1 summarizes the failure modes that can be detected by visual inspection (by naked eye), imaging, and electrical data analytic methods. Some failure types (e.g., soiling and partial shading) can be detected by several techniques, while other failure modes (such as module mismatch) can only be detected by one technique.

**Table 1:** Failure modes detected by visual, imaging and data analytic techniques.

TYPE OF FAILURE	VISUAL	IMAGING			ELECTRICAL DATA ANALYTICS
		IRT	RGB	EL	
Bending of the frame	✓		✓		
Bridged fault					✓
Broken/cracked cell	✓	✓	✓	*✓*	
Burn marks	✓	✓	✓		
Clamping	✓		✓		
Corrosion	✓		✓		
Degradation of the transparent conductive oxide (TCO) and edge degradation (for thin film)				*✓*	
Degradation rate					✓
Delamination	✓	*✓*	✓		
Disconnected cell and string interconnect ribbons		*✓*		✓	✓
Earth (ground) fault					✓
Ethylene vinyl acetate (EVA) discoloration (yellowing/browning)	✓		✓		
Glass breakage	✓		✓		
Hotspots		✓		*✓*	
Inverter shutdown					✓
Junction box (JB) failure	✓		✓		✓
Light-induced degradation (LID)		✓		*✓*	✓
Line-to-line faults					✓
Maximum power point tracking (MPPT) fault					✓
Module mismatch					✓
Open-circuit fault		✓			✓
Partial shading	✓	✓	✓		✓
Potential induced degradation (PID)		✓		*✓*	✓
Short-circuited PV modules in a string		✓			✓
Shorted bypass diode		✓			✓
Snail trail	✓		✓	*✓*	
Soiling	✓	✓	✓		✓
Soldering	✓		✓		
Welding defect in the joints	✓		✓		

Fault detection accuracies ranging from 83 % up to 100 % [3,26,83,[101], [102], [103]] were reported in the literature when using electrical data analysis methods for fault detection. The classification accuracy was reported to be between 81.70 % and 100 % [3,[19], [20], [21],^43^,^85^,^99^,^100^,^104^], when using electrical data characterisation methods for the diagnosis of open- and short-circuit failures, module mismatches, partial shading conditions, bypass diode failures, soiling and degradation. With regards, to imaging techniques, the detection and classification accuracies ranged from 83 % up to 99.91 % [25,26,[105], [106], [107]]. More specifically, IRT imaging processing resulted in fault detection and classification accuracies ranging from 92.80 % to 99.91 % and from 91.20 % to 99.80 % respectively for diagnosing fault modes (such as bypass diode failure, partially covered PV module, partial shading, short-circuit and dust deposition) [107,108]. The analysis of RGB images showed diagnostic accuracies ranging from 28.20 % to 98.59 % when categorising soiling, encapsulant delamination, glass breakage, gridline corrosion, snail trails and yellowing [25]. Results obtained from the processing of EL images showed accuracies ranging from 83 % to 94.50 % when diagnosing micro-cracks, partially broken cells, finger interruptions and shunt faults [26,105].

## 3 Requirements of image tecniques for PV failure diagnosis applications

To achieve precise failure diagnosis through image analysis, specialized instrumentation such as sensors or cameras, specific monitoring architectures involving data acquisition devices and configurations, as well as aerial systems, are essential. These components collectively facilitate the utilisation of UAV-based methods for failure diagnosis.

### 3.1 Requirements for image diagnostics

#### 3.1.1 Infrared thermography

**Fig. 8:**
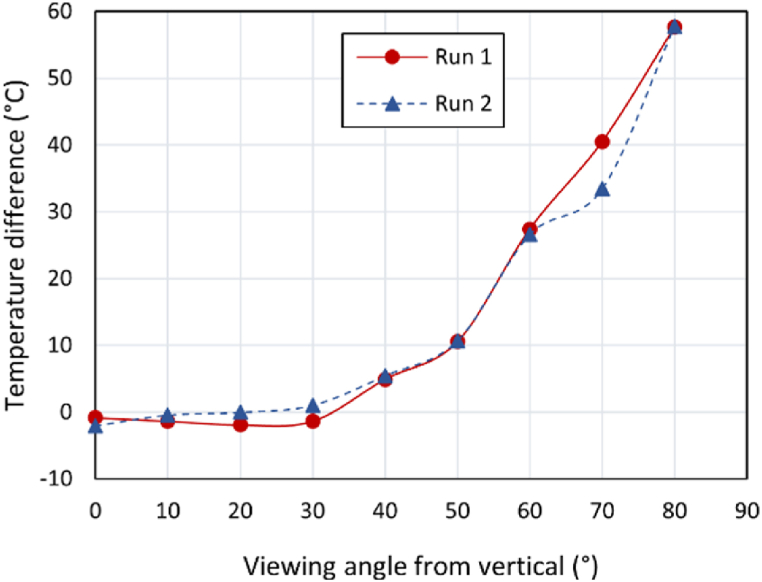
Temperature differences and viewing angle. Figure recreated from Ref. [109].

Generally, different factors and anomalies can affect the accuracy of the diagnosis using IRT images [65]. Such factors are linked to the used technique, failure type, material type, defect depth and excitation time. Other external factors may also affect the acquired measurements/images resulting to wrong temperature and emissivity variations, reflections, camera malfunctions, etc. Ball et al. in Ref. [109] investigated how the viewing of angle of a camera influences the temperature (see Fig. 8). The results showed a strong variation in measurements, especially for viewing angles in the range of 40–80°.

Image resolution is another key point for PV aerial thermographic inspection. The authors in Ref. [91] performed three thermal inspections (a traditional inspection and two aerial inspections at 30 m and 80 m, respectively) to a 3 MW PV plant. The detected anomalies from the three inspections are summarised in Table 2. The traditional inspection was the most comprehensive as it identified a total of 1132 distinct failures. Conversely, only 37.20 % and 23.10 % of the reported anomalies were detected by the aerial inspections at 30 m and 80 m flight height, respectively.

**Table 2:** Anomalies detected comparing three thermal inspections: manual, aerial at 30 m and aerial at 80 m [91].

ANOMALIES DETECTED	MANUAL	AERIAL 30 m	AERIAL 80 m
ΔT≥30°C	92	9	0
10°C≤ΔT<30°C	305	231	73
ΔT<10°C	738	181	202
Total	1132	421	275

Regarding the analysis of aerial IRT, the most important aspects include the resolution, thermal sensitivity, accuracy, lens, the corresponding field of view (FOV), radiometric functionality, frame and/or temperature range of the thermographic camera, stability of the system and maximum altitude [110]. Additionally, maintaining steady state conditions is necessary; a minimum of 600 W/m^2^ of solar irradiance, wind speeds within 28 km/h, a maximum UAV speed of 3 m/s, clean modules, accurate angle of view to prevent reflections, accurate emissivity, reflected temperature, relative humidity, and camera settings. Properly selecting the temperature range and calibrating the camera are also imperative [110].

Other considerations for acquiring thermal/IRT images are reported in the IEC TS 62446-3 [68], and the minimum requirements for PV inspection using IR cameras are summarised in Table 3. As such the IR camera should be mounted vertical to the PV module surface to prevent glass reflection. In cases the images cannot be taken perpendicular, the angle should be greater than 30°. Furthermore, drone velocity can affect negatively the image quality; when the UAV velocity is higher than 3 m/s, it can cause smearing [111]. Lastly, for the environmental conditions, a minimum irradiance of 600 $W/m^{2}$, a maximum wind speed of 28 km/h, clear sky conditions and no soiling are recommended.

**Table 3:** Minimum requirements for IR cameras [68].

a/a	FEATURES	MINIMUM REQUIREMENTS
1	Spectral response	2 μm to 5 μm (mid wavelength) or 8 μm to 14 μm (long wavelength)^1^
2	Temperature-sensitivity and calibration range (object temperature range)	−20 °C to +120 °C
3	Operating ambient air temperature range	−10 °C to +40 °C
4	Thermal sensitivity	NETD ≤0,1 K at 30 °C
5	Geometric resolution	1) PV module: max. 3 cm of the module edge per pixel^2^ 2) Electrical connections: The geometrical resolution (Real measurement spot^3^) has to match the smallest object area to be verified. (e.g., cell)
6	Absolute error of measurement	< ± 2 K
7	Adjustable parameters	Emissivity (*ε*), reflected temperature (Trefl)
8	Adjustable functions	Focus, temperature level and span
9	Measurement functions	Measuring spot, measuring area with average and maximum temperature
10	Calibration	The system for measuring (camera, lens, aperture, and filter): At least every two years, the thermographic camera must be traceably calibrated. Calibration must be documented. If the camera is not compliant (absolute temperature and/or temperature differences), it may be readjusted by the manufacturer.
11	Documentation	Storing the infrared picture along with all radiometric information to determine absolute temperatures. Non-radiometric pictures can only provide pattern and eventually temperature differences.
1 Cameras operating in the 2 μm to 5 μm wavelength should only be used for thermography of electrical BOS components, such as fuses. Given the transparency of glass in the range of 3 μm, using that range on PV modules can result in measurement inaccuracies. 2 On a 6-inch PV cell, 3 cm of edge per pixel equals 5 x 5 pixel. 3 For high-quality optics, the true measuring spot is usually defined as 3 x 3 pixel.		

When working with IRT imagery, the image resolution and ground sample distance (GSD) are highly relevant parameters for the fault detection procedure. The IEC TS 62446-3 [68] suggests that all PV module images should be captured with a minimum resolution of 5 × 5 pixels per cell. Gallardo-Saavedra, S. et al. in Ref. [91] proved that the GSD highly affected the fault detection capabilities; a GSD of 1.80 is required for identifying all thermal defects. Also, a common approach followed by PV plant operators for fault classification is the definition of temperature ranges for attending the severity [91]: a) severe faults for differences higher than 30 °C, b) medium severity faults for temperature values between 10 °C to 30 °C, and c) minor severity faults for temperature differences lower that 10 °C.

The resolution of regular cameras for RGB images must be substantially higher than that of an IR camera (usually at least 30 times greater). For an example, when inspecting PV systems with both a regular and an IR camera, a resolution of at least 9 M pixel and 640 x 480 pixel, respectively is considered to be suitable for diagnostics [68].

#### 3.1.2 Electroluminescence imaging

Camera requirements (i.e., type of sensor, resolution, sensitivity, spectral band, type of cooling, test conditions, size and weight) are a key point for EL inspection of PV plants. Different camera detectors have been used in the literature, with the most common ones being the charge coupled device (CCD) and the complementary metal oxide semiconductor (CMOS) [10]. Each camera detector has its own merits and limitations (for example resolution, exposure time, etc.). Commonly, CDD sensors are constructed using silicon-based materials, while the CMOS ones are made of silicon or InGaAs materials. Each sensor has its own quantum efficiency (i.e., the ratio of incident photon to converted electron), which depends on the material used (i.e., the spectral band of each material). Variations of sensor type and optimization procedures have yielded in several sensors/products with different sensitivity, resolution, cost and spectral response.

The selection of the EL camera type depends on a range of factors. It is usually depended on the available budget, required sensitivity and resolution [10]. For instance, CCD cameras often exhibit superior resolution at lower cost compared to CMOS sensors, making them a popular choice. Though, CMOS cameras are more sensitive in the emission spectra of the crystalline silicon and some thin film technologies [10]. Nevertheless, silicon CCD is a perfect fit for the emission spectra of CdTe, thus is usually the recommended camera type for such applications [10]. Table 4 summarizes the different classes and the typical parameters for EL cameras.

**Table 4:** General camera requirements [10].

CAMERA PARAMETERS	LOWER CLASS	MEDIUM CLASS	PROFESSIONAL CLASS
**Type of sensor**	CDD	CDD/CMOS	CDD/CMOS
**Resolution** (CDD) (CMOS)	<1Mpixel	1–5Mpixel 320×256	>5Mpixel 640×512
**Sensitivity** (dynamic range) (exposure time)	2500:1 >10s	5000:1 1–10s	10000:1 <1s
**Spectral band** (Si) (InGaAs)	0.30–1.10	0.30–1.10 0.70–2.60	0.30–1.10 0.70–2.60
**Test conditions**	Twilight/night	Twilight/night	Lowlight/night

Each camera used for capturing EL images has a certain resolution. Depending on the failure mode for detection, higher/lower resolution may be needed. Higher resolution images can be obtained from pictures taken from a longer distance, depicting more modules per image. The sensitivity represents the camera's performance, including the ability to distinguish between the signal and the surrounding background noise. The signal-to-noise ratio (SNR) can be increased by enhancing the sensor's quantum efficiency (spectral band) or/and by reducing noise sources (cooling). It is important to note that the selected camera sensor is sensitive to the material's emission spectra (e.g., c-Si, CdTe, CIGS, etc). The sensor's ability to respond to the signal refers to its quantum efficiency [10]; a sensor is suitable/applicable when it has an overlapping band with the testing material. Additional coatings or lenses can be used to further boost the quantum efficiency. The camera should be cooled for reducing noise (which increases with longer exposure times). Exposure durations exceeding 10 s require deep cooling [10]. The cooling demand is reduced when the exposure durations are <1 s. Most of the EL systems are exclusively operational during nighttime or at low light conditions. Nevertheless, systems designed for use in daylight offer increased operational availability but require more sophisticated equipment and image processing [10].

The weight and size of the sensor are also crucial parameters to consider depending on the application. For instance, in UAV applications, larger and heavier cameras are not suitable [10]. Moreover, stationary EL cameras employed within portable configurations or smaller handheld systems are utilised for onsite ground-level inspection. Depending on the power supply size, an inspection of individual modules or entire strings could be achieved. A connection of the power supply and the string to a multi-box with multiple inputs and a remote-controlled switch could improve the recording rate and hence the inspection productivity. Other applications may demand employing a remote-controlled pivot arm mounted on a telescopic tripod, such as roof-mounted systems. Due to the EL cameras' inability to autofocus at night, distance sensors are recommended to determine the distance between the camera and modules to change the camera's focal length accordingly. In summary, the use of manual procedures could increase productivity of around 0.20 MW_p_ for the inspected PV systems per night [10].

On the other hand, EL cameras installed on UAV can be used to carry out aerial inspections. Bearing in mind that higher sensitivities and shorter exposure times are needed, not all EL cameras are suitable for aerial applications [10]. The most suitable cameras for such application are the mirrorless full-format ones. As stated earlier, the UAV should have a distance sensor and a remote-controlled focus length for ground-level inspections. Due to the limited battery life, a power supply and multi-box combination are also strongly recommended. Additionally, the camera's angle should be adjustable, and the UAV should have a global positioning system (GPS). With aerial inspection, the productivity can be increased up to 1 MW_p_ of inspected PV systems per night [10].

The module's orientation in relation to the camera aperture introduces some intrinsic human-induced inaccuracy into EL measurements carried out in laboratories. The authors in Ref. [10], presented a process for converting unaligned raw images into uniformly orientated ones. As a first step, the noise in the image had to be removed. A filter (e.g., median filter) that preserves edges and does global smoothing was utilised for detecting features in the image. Given the range of available techniques, numerous methods can be utilised to determine the active cell region. The convex Hull approach was utilised to entirely enclose the active cell sections for ease of implementation in Ref. [10]. The goal of this approach was to identify the smallest convex set that can contain all of the image's non-zero regions. Additionally, the method was insensitive to outer cell cracks or gaps between cells. In particular, the identification of the edged was obtained using periodic vector slices of the image, discovering the locations when the vector values switch from 0 to 1 or vice versa, and then fitting a linear regression line to those locations. The proposed approach could be implemented easily and was effective for pipelining large image datasets.

Many panels may be inspected using PV imaging equipment. Automated image analysis is needed to detect, measure, and report defected PV panel using tens of thousands of panels images. However, one of the primary causes of errors in automatic analysis of images is the module image distortions due to the process of acquisition [5].

#### 3.1.3 Unmanned aerial vehicles

UAV with integrated thermal and RGB cameras have been used to support plant inspection and PV fault detection [74,75,112,113]. Many studies in the literature involve the application of different UAV and imaging sensors. For example Lee, D. H. et al. in Ref. [114] used the DJI Inspire 2 commercial drone equipped with a FLIR Vue Pro R and a DJI Zenmuse X5s camera for thermal infrared and optical image acquisition. Zefri et al. [74] used two models of SenseFly drones; (a) the Albris eMotion 3 quadcopter with the on-board RGB and thermal cameras and (b) the eBee fixed-wind UAV equipped with a Canon IXUS 127HS and a ThermoMAP RGB and thermal cameras, respectively. Henry et al. in Ref. [72] developed a custom-build UAV platform equipped with a FLIR Vue Pro R and a Logitech C270 camera for thermal and RGB image acquisition. Two main drawbacks were the remote control and no localization of faulty PV modules.

An aerial inspection platform for fault diagnosis of PV parks should present the following unique capabilities: accurate geolocation, autonomous operation, and on-board processing. Accurate geolocation also constitutes an important aspect for fault diagnostics and O&M [72]. For autonomous operations, both single but also swarm type solutions can be used for efficient PV plant monitoring [115]. A fully autonomous collaborative scheme can be developed, where the UAV will work together and adapt their flight plan to cover possible gaps in full area coverage. The establishment of on-board processing capabilities will constitute another significant novelty for UAV-based platforms. Apparently, the computational efficiency of the implemented processing algorithms will play an important role in the accomplishment of this objective.

To sum up, the design of the UAV platform is strongly related to the payload, the specifications of the ground sampling distance, the size of the plant to be monitored and the environmental conditions. Table 5 provides the physical dimensions along with a roughly estimated cost of the candidate sensors to be installed on a UAV platform. Another important criterion for the design of a UAV platform is the interoperability and communication protocols among different modules: flight controller, platform stabilization, heading accuracy, autonomous operations, etc.

**Table 5:** Main sensor that can be integrated to the UAV for fault diagnosis in PV systems.

SENSOR	TYPE	WEIGHT (gr)	DIMENSIONS (mm × mm × mm)	EST. COST (€)	REF.
FLIR T620 (Discontinued)	IRT	1300	143 × 196 × 94	17600	[116]
FLIR A310	IRT	700	170 × 70 × 70	10000	[116]
FLIR TAU 2	IRT	<70	44.50 × 44.50 × 30	7400	[116]
FLIR TAU 2640	IRT	<70	44.50 × 44.50 × 30	6500	[116]
FLIR A35	IRT	200	106 × 40 × 43	5216	[116]
FLIR Vue Pro R	IRT	113	57.20 × 44.50	4849	[116]
DJI Zenmuse XT	IRT	270	103 × 74 × 102	10000	[117]
DJI Zenmuse XT2	IRT	270	103 × 74 × 102	6800	[117]
DJI Zenmuse X3	IRT	215	75 × 95 × 105	459	[117]
Optris PI 450	IRT	320	46 × 56 × 76	4000	[118]
Fluke Tis10	IRT	720	267 × 101 × 145	1350	[119]
Thermoteknix MicroCAM 4	IRT	30	36 ∅ × 24.50		[120]
Workswell WIRIS Pro	IRT	<430	83 × 85 × 68	17000	[121]
ThermoMAP (including the whole SenseFly eBee X)	IRT/Drone	5000	750 × 500 × 290	13500	[122]
Yuneec CGOET	IRT	275	81 × 108 × 138	2000	[123]
GoPro Hero 3	RGB	163	41 × 58 × 30	190	[124]
Nikon 1 V1 HD	RGB	383	113 × 76 × 44	600	[125]
DJI Zenmuse X5s	RGB	461	140 × 98 × 132	2199	[117]
Canon IXUS 127HS (Discontinued)	RGB	135	93.20 × 57 × 20	260	[126]
Logitech C270	RGB	75	73 × 32 × 67	30	[127]
FLIR A6260sc	EL	2500	216 × 102 × 109		[116]
Raptor Photonics Owl 640 SWIR	EL	282	42 × 42 × 67	10000	[128]
DJI S1000	Drone	4200	460 × 511 × 305	1200	[117]
DJI S900	Drone	3300	460 × 450 × 360	3600	[117]
DJI Matrice 100 (Discontinued)	Drone	3400	Wheelbase 650 ∅		[117]
DJI Matrice 300 RTK	Drone	6300	810 × 670 × 430	13800	[117]
DJI Inspire 2	Drone	3440	427 × 317 × 425	3400	[117]
Nimbus EosXi	Drone				[129,130]
Nimbus PLP-610	Drone	2800	980 ∅ × 250		[129,131]
DaVinci Copters ScaraBot X8	Drone	<5000	Central distance 500 ∅		[132]
Skyrobotic SR-SF6	Drone	5500	1100 × 1100 × 360		[29,133]

UAVs provide various benefits and unique opportunities for field PV applications. This can be attributed to the latest developments in aerial technology, sensors, and control systems which support UAV and make them an appropriate tool for inspecting and monitoring PV systems [64]. Extensive coverage, exceptional flexibility, low weight, autonomous operation and fast speed can be achieved when using UAV-based systems [76]. However, some challenges may arise when developing such systems, deteriorating the effectiveness and the autonomy of UAV's. Such challenges are concerned with the localization of the drone (where the precision of the geolocalization is not sufficient to allow flying at low altitudes), autonomous flight duration and flying paths over the plant to avoid overlap and empty areas between the PV strings, acquired images resolution and quantification of the impact of each fault's pattern on PV performance degradation [64,72,115,[134], [135], [136], [137], [138]]. Towards tackling these challenges, vision-based control laws were suggested to track PV panel rows based on PV modules' edge detection [134,136,139], while different techniques were also proposed to extract the plant's boundary via computer vision techniques and compute the UAV path over the plant [135,138].

In this context, Lofstad-Lie et al. [64] presented an aerial IRT defect inspection procedure for PV power plants utilizing a two-step autonomous flight strategy to decrease the inspection duration (approximately 60 %) and associated costs. The first step involved a fast-high-altitude flight for detecting possible defect location. At the second step, an optimised flight path at lower altitude was calculated based on heuristic ant colony optimization (ACO) algorithm to inspect the locations detected previously with defects. This enabled sufficient resolution to be acquired for defect classification. The inspection method was implemented using an DJI Matrice 100 drone and an Optris Pi 640 thermal camera and proved to be beneficial for plant geometries representative of high latitudes.

Zhang, C et al. in Ref. [140] presented a method for UAV path planning utilizing a heuristic crossing search and rescue optimization algorithm (HC-SAR). A real-time path adjustment strategy and cubic B-spline interpolation were also used to straighten and smooth the UAV flight path, respectively. Two- and three-dimensional simulated environments with different threat zone were utilised for testing and validating the HC-SAR approach. Their approach managed to overcome the problem of slow convergence speed when using the search and rescue optimization algorithm (SAR). Lastly, the results indicated that HC-SAR could converge fast and identifying a safe and efficient path, outperforming differential evolution (DE), SAR, squirrel search algorithm, ant lion optimizer (ALO) and salp swarm algorithm (SSA) in all the test cases.

Moradi Sizkouhi, A.M. et al. [135] proposed an automatic boundary extraction for large PV installations based on fully convolutional network (FCN). In their study, RGB aerial images from different PV plants around the world were used. The proposed FCN procedure was based on a Mask R–CNN architecture with a modified fine-tuned VGG-16 model to accurate detect boundaries. The FCN achieved accuracies of 97.61 % and 96.93 % for training and testing, respectively.

Xi, Z. et al. [134] presented a vision-based inspection strategy using an autonomous UAV, suitable for large-scale PV farms. The inspection strategy consisted of three main aspects, the mission areas acquisition, line detection and calculation, and velocity controller. The first aspect was realised by initially gaining the boundary information through design drawings, GPS, and aerial images along with geographic information system (GIS). Then subsequent treatment was applied to derive the “Regional Polygons” of the PV plant and determine a start point chosen based on the northern or southern vertices of each “Regional Polygons”. The second aspect was crucial for the velocity control of the UAV, and it was performed in three steps. First the RGB images were converted in Hue, Saturation, and Value (HSV), and the edge of the PV string were extracted using Canny edge. The Hough transformation was implemented to identify straight line and lastly, the slope of the edge lines and offset from the image was estimated. Finally, the velocity during tracking and turning procedure was controlled in order to minimize deviation from image centre and ensure tracking of PV strings.

Pérez-González, A. et al. in Ref. [138] proposed coverage path planning (CPP) methods for automatic UAV flight path in PV plants. A DL server to segment the region of interest (RoI) of each PV plant image, and three CPP techniques were used and compared. In particular, the CPP methods used were the boustrophedon exact cellular decomposition, grid-based spanning tree coverage and wavefront coverage. The performance of the energy consumption in each of the CPP method was also evaluated using two different UAV. The results showed that the boustrophedon exact cellular decomposition and grid-based wavefront coverage were the most effective approaches.

The movement of the UAV as well as the preferred settings of an IRT sensor to assure the correct acquisition and reading of IRT images during aerial inspections was investigated in Ref. [141]. Several crucial parameters regarding the operation of the UAV-IRT system were outlined, including the UAV's flight height and speed, as well as the association between the incidence angle and the emissivity. For the latest, as it depends on the UAV-IRT system and not fully on the expertise of the operators, the authors suggested a semiempirical equation to support PV diagnostics. These equations provide ease of correcting the thermal map in post-processing and avoidance of radiometric errors due to the distance of the UAV. Finally, to avoid blurriness in the acquired images and fixed resolution, a maximum speed of 3 m/s was suggested. Nevertheless, it should be noted that even if the fast acquisition is one of the primary benefits of this method, a large amount of raw data was produced, requiring more sophisticated processing tools [139]. The processing procedures can involve a more manual approach, processed by an expert user, or an automatic one, which consists of computational techniques for image post-processing [139,142]. The automated approach is a fast and an efficient way of processing enormous amount of data, making the entire process of UAV acquisition competitive. Different analysis techniques can be used to support the UAV image acquisition, with the most advanced ones focusing on the health status of each module, exploiting deterministic algorithms [143] or AI techniques [38,144].

Lastly, another factor that needs to be addressed is the resolution needed for post-processing. This is an important factor for designing a correct flight plan for UAV inspection. Based on the IEC TS 62446-3, a resolution of 5 × 5 pixels per cell within a 15 × 15 cm area is regarded as the minimum required for classifying module failures [68]. Thus, a balance between the altitude of flight and the precision of the UAV sensor has to be made to reach the required resolution [91], with the low flight height usually being preferred [141].

#### 3.1.4 Monitoring systems

Monitoring systems speed up the O&M of a PV plant, therefore choosing the right means and platforms for the use case can be a major investment in terms of time and cost. A wide variety of applications can be found in the literature using various approaches and concepts, that are adapted to the data, infrastructure and available resources [[145], [146], [147], [148], [149]]. For data collection and processing, three different representation approaches are used: (a) visual (from thermography-based detection), (b) graphs (based on measurements and estimations), and (c) mixed representation [6].

**Fig. 9:**
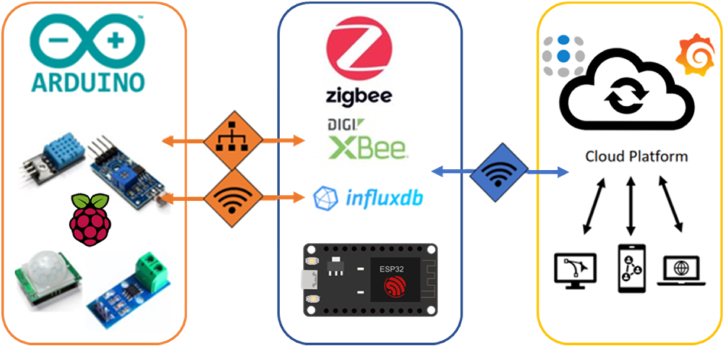
Diagram of the monitoring system architecture.

Monitoring systems architecture consists of three different layers (see Fig. 9) [6,150]. The first layer is the reading point of the PV system consisting of the sensors, microcontrollers, cameras and all the hardware components necessary to obtain the raw data/images. The second layer consists of the gateways and connection points between the first layer and the server side or database with the necessary configuration. Finally, the third layer consists of the framework used for monitoring and control, which can be developed from standalone applications to cloud service applications, where the processing of stored data can be tackled server-side, and algorithms for fault and malfunction detection can be applied.

Regarding data acquisition, two different data sources are used; (a) the numerical and environmental data obtained by sensors of the system and (b) the obtained images (e.g., IRT, RGB and EL images). For the first case, the use of IoT technology predominates, where its principle is the connection between sensors and devices in a common network of wired or wireless nodes. The wireless connection leads to a simplified data collection process by all the sensors in a common area. Such process requires the aforehand installation of devices connected directly to the internal systems of the monitored PV plant (e.g., installation of voltage and current sensors for measuring data at the PV string level).

Raspberry Pi boards and Arduino microcontrollers (such as ESP32) are usually used to collect and process the information gathered by multiple sensors. These devices are also used to establish a two-step communication [[145], [146], [147], [148], [149]]. The first stage involves the communication between the sensors and the microcontroller through an internal network, and the second one concerns the grouped data uploading to the storage site, either by ethernet or wireless connection. Regarding the image acquisition, these are acquired using fixed cameras, or cameras embedded on UAV. The latter is proved to be a revolutionary tool for the O&M sector [9]. The images are then stored by a manual process (i.e., manually extracting the images from the camera and storing them to designated site) or by using 4G/5G technologies which can transfer and store the captured images to the cloud service.

The third layer is dedicated to the processing of the data, representation and visualization of the results. The most common sensor-based data for representation include power, current, voltage, temperature, humidity, and irradiance measurements. These measurements (stored in the database) can be easily represented in different graphical formats such as charts, panels, metrics, indicators, gauges, graphs, etc. In some cases, specific software tools, databases and dashboards (e.g., Grafana cloud platform and InfluxDB [145]) can be used for better representation and to allow access points to users using service set identifier (SSID) and passwords through any device. An integrated dashboard may also be included, depicting all the information about the system health-state, and sending automatic alerts and notifications in case of faults.

An architecture oriented to the infrastructure as a service (IaaS) model was presented in Ref. [146] and compared with different alternatives such as Eucalyptus, Open Nebula and OpenStack. Based on the results from the comparative analysis, OpenStack was used along with a PostgreSQL database in the cloud, and the Arduino Mega for processing the data. The interface allowed the selection of a single generator, loading multiple variables in different formats. The graphical analysis of the monitored variables facilitates the study of the energy performance of the PV modules, providing information on the generation values through the calculation of the produced power in each time period. A similar architecture was presented by Li et al. [151] for online monitoring of PV systems. The architecture used ML techniques for fault detection, while the operating indicators of PV system were uploaded to the data gateway using a ZigBee network. Other tools were also added (e.g., tools to acquire 10.13039/100011109GPS information, FFA-SVM (firefly algorithm - support vector machine) model, etc.), and all the data were uploaded to ubidots platform (Open IoT Platform) via Wi-Fi providing at the end an open-source platform.

## 4 Integration of UAVs with image approaches to support PV plant diagnosis

### 4.1 Infrared thermography

**Fig. 10:**
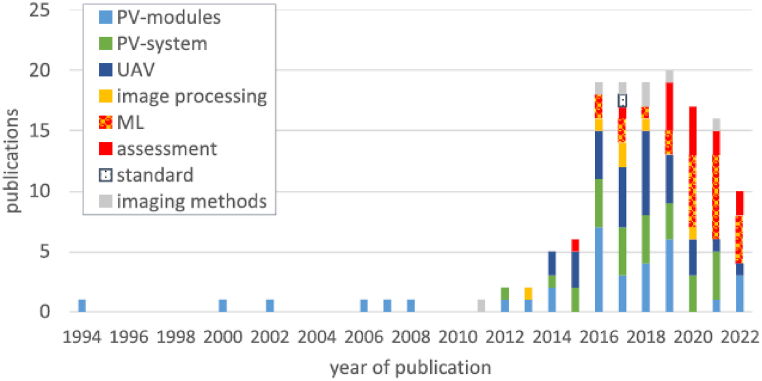
Overview of the 142 publications on thermography-related inspection of installed PV systems [9].

Over the past decades, IRT have attracted an increased attention for inspecting installed PV systems with over 100 publications focusing on inspections using IRT images as shown in Fig. 10 [9].

Dunderdale, C. et al. in Ref. [67] proposed two part models for PV defect classification between 4 different errors. The first part was a feature-based methodology consisting of acquiring data, extracting features using SIFT descriptors, feeding them to a ML algorithm, and then using the ML algorithm to classify modules as defective or non-defective. The second procedure was based on DL using two CNN architectures (i.e., MobileNet and VGG-16) for defect classification. The dataset was composed of 798 thermal images, 398 with defective panels, 400 non-defective. The dataset of the defective panels consisted of different hotspots patterns as follows: 32 % block, 22 % patchwork, 22 % single, 21 % string, and 3 % soiling. For the image acquisition process, the FLIR Tau 2640 thermal camera was utilised under operating conditions. The feature-based methodology achieved classification accuracies up to 91.20 %, while the DL model for differentiating defects achieved an accuracy of 89.50 %.

Mellit, A. in Ref. [106] proposed an embedded solution based on IRT images and deep convolutional neural networks (DCNN) that could identify PV modules defects. The solution was based on two DCNN-based models, a binary classifier and multiclass classifier for the fault detection and classification stage, respectively. Four common faults (i.e., bypass diode, dust deposition, short-circuited PV module and partial shading) were investigated and the results revealed a high diagnosis accuracy of 99 %, while the classification accuracy was 95.55 %. For real-time diagnosis, the developed models were embedded into an edge device (Raspberry Pi 4) which in connection with a GSM (SIM808) module could alert operators for the PV installation state through SMS, email and an LCD display showing the results.

Kellil, N. et al. in Ref. [107] developed and compared a CCN and a fine-tuned model based on VGG-16 for PV fault diagnosis using IRT images. A binary classifier was used for fault detection, while a multiclass classifier was used for fault classification. The classification accuracy of the developed models was tested on 5 fault modes (i.e., bypass diode failure, partially covered PV module, partial shading, short-circuit and dust deposition). The results showed that the VGG-16 model achieved higher accuracy (99.91 % and 99.80 % for fault detection and classification, respectively) than the small DCNN model (98.39 % and 91.63 % for fault detection and classification, respectively).

The recent diffusion of UAV, incorporating a thermal camera, facilitate monitoring work usually carried out from the ground or lifting platform with handheld cameras by operators. This process relies on human labour, and result in a time-consuming and labour-intensive method, also susceptible to dangerous situations and accidents for operators and PV plants.

Pierdicca, R. et al. [69] demonstrated that DL architectures can be employed for the detection of damaged PV cells. The training of the detection algorithm was performed using DCNN and specifically, VGG-16 network architecture with input augmentation and no pre-processing on a dataset of labelled thermal images. The dataset consisted of 3336 thermal images taken by a Skyrobotic SR-SF6 drone with an FLIR TAU 2640 IRT camera at a ground-based PV plant of 66 MW in South Africa. Within the dataset, 811 of the images had an anomaly (i.e., a faulty PV module). The analysis was performed using two datasets, (a) the unbalanced dataset which used the whole dataset of 3336 IRT images and (b) the balanced dataset which consist of the equal amount of 811 for both normal and anomaly images. The best detection accuracies were achieved when using the balanced dataset with data augmentation (rotated images), with a 76 % precision and 75 % for both recall and F1-score.

Nie, J. et al. in Ref. [70] proposed a convolutional neural networks (CNN) based approach, defining that the task of object detection is more complex compared to hotspot classification specifically for large-scale PV plants. The basic methodology applied arises from the pre-processing algorithm for the extraction of individual modules, then for their subsequent binary classification. The proposed method used UAV IRT images from a PV power plant in Datong, Shanxi Prov (4000 images 70-20-10). Based on a 70:20:10 % training, validation and test sets, it yielded an accuracy of 95 % for hotspot diagnosis.

Another study presented by Hwang, H. P. C. et al. [23] involved the design of a hybrid scheme based on three learning methods to improve the detection malfunctions such as hotspots, PID and open-circuit in PV modules using IRT images obtained by a UAV. In particular, the hybrid scheme included a DL algorithm, image pre-processing (i.e., gamma-correction, thresholding, Sobel, Canny), and a ML algorithm. For the analysis, 684 images from the roof of the Industrial Technology Research Institute in Hsinchu, Taiwan were evaluated based on a 76:24 % training and test set distributions. The results of the hybrid scheme with three models yielded an average classification accuracy of 99.20 %.

Herraiz, Á. H. et al. [27] suggested a methodology for online detection of fault PV conditions based on a combination of thermography, GPS positioning and CNN analysis. Specifically, a R–CNN based methodology, i.e., a CNN type of method for object detection and semantic segmentation, was developed to recognize panels’ region of interest (ROI) and detected faults inside squares based on relative hot areas. The analysis was performed using 800 images, of which 320 included hotspots that were captured by a DJI S900 drone with a WIRIS WORKSWELL IRT camera. The suggested method achieved an accuracy of 99 % and a precision of 91.70 % for detecting and localizing relative hot regions.

Manno, D. et al. in Ref. [71] have built a DL strategy for the classification of thermographic images obtained both by ground-based operators and UAV. In their study they used a CNN model and investigated different cases, e.g., different number of images in a dataset, resolution and operator/UAV views, pre-processing procedures (resize, RGB, grayscale, thresholding, Sobel Feldman, etc.), and algorithm architectures. The accuracy varied from 93 % to 99 %, with the best one obtained by the 1000-image dataset. However, this method does not offer real-time fault diagnosis according to their severity.

Dotenco, S. et al. in Ref. [108] proposed an image processing pipeline designed to automatically evaluate PV power plants. The first step was to identify the individual modules within an IRT image. To achieve this, a statistical approach was used, that incorporated normalization, thresholding, orientation calculation of the PV modules, correction and refinements techniques. Then statistical tests (i.e., the Grubbs' and Dixon's Q-tests) were used to detect the defected modules. Specifically, four feature sets (i.e., module medians, grid cell medians, histogram skewness, and vertical projection) were examined to detect and classify the defects (i.e., overheated modules, hotspots, and overheated substrings). For the analysis two PV plants of approximately 7 MW_p_ located in Bavaria, Germany, were used. IRT images were captured using DaVinci Copters ScaraBot X8 with embedded an IRT camera Optris PI450 and a RGB camera GoPRo Hero3+. The accuracy was evaluated using 24 IRT images, achieving a F1-score of 92.80 %. On the other hand, for the defects' classification, 37 hand-labelled IRT images (1544 PV modules) were used, yielding a F1-score of 93.90 %.

Pierdicca, R. et al. in Ref. [29] implemented an automatic system “solAIr”, which was based on DL for the detection of anomalies and geopositioning of damaged cells in PV thermal images obtained by a UAV device. The anomaly detection architecture was based on the Mask R–CNN, trained, and evaluated on thermal images. Additionally, they did a comparison and a performance evaluation of various DNNS methods, such as UNet, FPNet and LinkNet. For the PV inspection and the image acquisition, the Skyrobotic SR-SF6 drone with embedded a radiometric Flir Tau 2640 camera was used. The evaluation of the results was based on the Jaccard index and Dice coefficient. UNet was the best performing method, resulting to Jaccar and Dice of 0.70 and 0.80, respectively. The annotated dataset used in their work was shared as a publicly available dataset. The proposed methodology lacks in the sense of fault classification, as it could classify each pixel as either a damaged cell or as a background.

Segovia Ramírez, I. et al. in Ref. [148] presented a fault diagnosis approach using multilayer perceptron (MLP) neural networks based on thermographic analysis, while employing a different condition monitoring system (CMS). The CMS consisted of an IRT sensor (model SI-131) with a data acquisition wireless system embedded in the UAV DJI S900, and it provided simple data that can are suitable for real-time analysis in IoT platforms. For the analysis, IRT images were utilised of 5 fault classes (i.e., no fault, hotspot/broken cell, fault cell, open-circuit, bypass diode and polarization). The images were captured under four scenarios: at 0° and 60° according to the horizontal plane of the PV panel and 2 days at different temperatures (at 22 °C and 18 °C). Fault detection and classification accuracies of 100 % and 96 %, respectively were achieved by the proposed method. The authors validated their system by using an IRT camera and other radiometric sensor as well as utilizing ANN and classification learner algorithms, obtaining similar results (100 % fault detection accuracy and 94–95 % classification accuracy).

Zefri, Y. et al. in Ref. [152] developed an encoder-decoder U-Net based semantic segmentation model for detecting and geolocating overheated PV modules using orthorectified thermal IRT UAV imagery. For the analysis, IRT images from 31 different PV installations (515 images) were acquired using Zenmuse XT (focal length of 13 mm) thermal camera, embedded on a DJI M210 V2 drone. Additionally, the image acquisition was performed at 40 m height with 85 % overlaps. Post-processing of the images was performed by applying a SfM-MVS pipeline of three steps along with augmentation techniques (flipping, shearing, scaling and rotation) to increase the original images by a factor of 10. Then, a random 85:15 % training and test set approach was used for the U-Net model, yielding a mean intersection over union (IoU), also called the Jaccard index, of 0.90 for detecting overheated modules.

Finally, Du. B. et al. in Ref. [153] proposed an eddy current thermography (ECT) system in an attempt to improve the fault diagnosis efficiency for Si PV cells. The thermography sequence processing for defect feature extraction was based on the principal component analysis (PCA), independent component analysis (ICA) and nonnegative matrix factorization (NMF) algorithms. For the classification of defects, a comparative analysis was performed between the LeNet-5, VGG-16 and GoogleNet models, and then benchmarked with other traditional classification methods, such as SVM and ANN. The findings demonstrated that the GoogleNet model yielded a high classification accuracy (average 97.90 %) when using first defect feature extraction, for defects such as broken edge, surface impurity, scratch, crack, hotspot, and large area damage. However, these results were extracted by using only silicon PV cells under an experimental setup.

### 4.2 Visible – RGB imaging

Espinosa, A. R. et al. in Ref. [25] presented two different classification CNN-based models for 2 (fault and no-fault) and 4 (breakage, shadow, dust and no-fault) output classes. The analysed images were RGB, and the dataset consisted of 345 images of solar panels at a 200x200 size, where the background and panel distinction were manually labelled with 0 and 1 respectively. The detection stage was performed using a CNN for semantic segmentation using 145 images as a training set. Furthermore, the classification was performed using the remaining 200 images based on a CNN model that classified breakages, shadows, dust and no-fault (50 images per class). This method yielded an average fault classification accuracy of 75.40 % for the 2 classes, and 65.70 %, 69.20 %, 41 % and 28.20 % (for the no-fault, dust, shadows, and breakages, respectively) for the 4 classes case.

Li, X. et al. in Ref. [154] developed an algorithm to address the limitations of R–CNN when confronted with multiple defects (i.e., dust shading, glass breakage, encapsulant delamination, snail trails, gridline corrosion and yellowing) analyses of PV modules with RGB visible images. First, a three-phase algorithm for abnormal region extraction consisting of (a) the edge detection with Kirsch operator, (b) background elimination with Hough line algorithm, and (c) interference elimination by the morphological opening operation was developed. Then, a CNN was suggested for extracting a feature vector from core area of the abnormal region. Finally, a multiple classification SVM (MC-SVM) was developed for defect classification at module level. The image acquisition was done by employing an DJI Matrix 100, armed with an optical single-lens reflex camera Zenmuse X3. Images from tens of PV plants, of total 1180 MW_p_, were captured to create the test dataset. The resulted average accuracy for the six defect types was 95 % (minimum value) and 97.20 % (mean value).

### 4.3 Electroluminescence imagery

During the past years, the PV community has been using EL imaging for defects detection. However, traditional visual inspection of EL images is required, which is a time-consuming and a costly task. Proper experience and examination of the obtained EL images are thus needed, and visual examination is only possible at lab/small-scale. For large-scale installations, automated detection techniques are of vital importance. Moreover, the increasing production line of PV modules and the world's growing interest in PV, made automated inspection process mandatory.

In this domain, Akram et al. in Ref. [83] presented an automated method for identifying defects in PV panels utilizing EL images. The approach included the use of light CNN architecture for diagnosing defects in EL images. The methodology yielded an accuracy of 93 % when applied to an open-source EL database [[155], [156], [157]], while using low computational power and accomplishing almost real-time speed (approximately 8.10 ms per image).

Parikh et al. [86] investigated different methods for improving the quality of an image and determining if the improved image offers more useful diagnostics for accurate detection of micro-cracks and fractures. The results showed that averaging aids in enhancing the SNR value. Also, subtracting the background from the test averaged EL image improved the image quality and the efficiency of micro-crack detection.

Demirci et al. [105] proposed an automated framework for detecting and classifying defects based on EL images. In particular, the deep feature-based (DFB) method extracted the image features through DL neural networks and then categorised them through ML methods (i.e., SVM, k-nearest neighbourhood, decision tree, naïve Bayes and random forest). Drawing from the identification of optimal features extracted from diverse layers within deep neural networks, classification scores ranging from 90.60 % to 94.50 % were obtained.

Tang et al. [26] suggested a DL method for classifying automatically defective PV panels using EL images. A data augmentation method was utilised to produce high-resolution EL images. A CNN model was then developed using the dataset generated by the combined data augmentation technique for training. The proposed model resulted to an accuracy of 83 % for detecting healthy, micro-cracks, finger-interruption and breaks in EL images. Different case studies (i.e., performance comparison with different data augmentation techniques, existing methods and different parameters selection, such as the impact of CNN depth, stochastic pooling and number of kernels) were carried out for evaluating model's performance. The case studies demonstrated that the proposed methodology performed better than other methods (i.e., MobileNet, ResNet50, InceptionV3 and VGG16).

Tsai et al. [158] proposed a self-reference scheme based on the Fourier image reconstruction technique for detecting defects from EL images. The obtained results showed that the developed method performed well for detecting small cracks, breaks, and finger interruptions. It was evaluated on 323 EL sub-images of solar cells (308 defect-free and 15 defective samples) and managed to correctly identify each fault in all the defective samples and no faults in the defect-free samples. Even though this method was based on the Fourier transform, it can also be applied to a small sub-image (with an average computation time of 0.30 s for a solar cell image of 550 × 550 pixels).

Mathias et al. in Ref. [89] develop a methodology for detecting micro-cracks in solar cells using EL images. Specifically, after pre-processing EL images to separate the individual solar cells from the PV panel, the authors used discrete wavelet transform (DWT) and stationary wavelet transform (SWT) for extracting textural features. Those features were then utilised for classifying PV cells into cracked and non-cracked ones employing SVM and back propagation neural network (BPNN). The classification accuracy was 92.70 % and 93.70 % for the SVM and BPNN, respectively.

An alternative study was presented by dos Reis Benatto, G. A. et al. in Ref. [159]. A drone-based daylight EL system was developed to acquire EL images during high solar irradiance. The initiative behind this study was to overcome the limitation of practicing the EL technique only indoors, or outdoors from dusk to dawn. This demonstrates the further potential of EL for utility-scale inspections (to be addressed in future research) and for the development of electrical biasing tools to make outdoor EL imaging fast and efficient. The results showed that EL images reached representative quality ${SNR}_{AVG}$ of 4.60. Even though the obtained quality is lower when compared with indoors and stationary daylight EL, it is adequate to identify disconnected cell regions.

Lastly, it is worth mentioning that measuring the power simultaneously to the EL images or alternatively predicting the power directly from the EL image can be an advantage. In this context, Buerhop et al. in Ref. [87] applied ML techniques to EL images for power prediction. The error of the predicted value with respect to the measured module power at MPP at standard test conditions, were evaluated for the models at differing degrees of distortion, such as lab conditions and, also, under varying outdoor conditions and differing image qualities. Fairly accurate results for EL images recorded in the lab and in the field resulted to a mean absolute error (MAE) of 4.60 W.

### 4.4 Combination of methods

As previously discussed, each technique has its limitations and merits. Thus, the integration of several techniques could enable additional capabilities to detect, classify, and localise a wider range of failure modes in a PV system. In this domain, Zefri et al. in Ref. [74] used both thermal and RGB photographs to perform the orthomosaics of the areas to be analysed. The proposed methodology was used for automatic hotspot detection. It consisted of superposition of thermal and RGB orthomosaics, PV string extraction delimiting them with polygons and interest points extraction by specifying a threshold for hotspots characterization. In the final representation, the defects were highlighted on an orthomosaic of the PV plant. The extracted hotspots based on the methodology of the authors was validated based on inspection performed by SenseFly team, revealing that both inspections detect the exact same hotspots.

By combining different data sources (e.g., electrical signals and images, such as EL, RGB and IRT), it is possible to obtain monitoring tools that provide much more information to users. In particular, Piccinini, F. et al. [160] combined aerial and terrestrial data to be managed in a GIS environment. In their study, aerial data were taken using a UAV drone, collecting RGB images to build an orthophoto of the PV system and used it as an interactive map in the GIS application. In addition, thermal photos were captured and reviewed using ThermoViewer. On the other hand, ground data were acquired with I–V curve tests. The final check consisted of verifying whether the power supplied was within the tolerance declared by the manufacturer. The system could highlight overheated components from the thermal inspection or the spatial distribution of the I–V curve. The results of the proposed GIS-based system reside in its capability to generate a comprehensive, adaptable, and interactive map of the PV systems offering the necessary information for enhancing the efficiency of O&M procedures.

In [11,161] a similar approach was carried out by implementing a GIS application, in which a data model represented individually each of the PV modules with their associated measurements and images, and on another side, the defects located for their individual positioning on each module. The GIS application allows applying filters to the modules to graphically represent the values of their modules by applying customised colour maps.

Mobin, O. H. et al. in Ref. [162] studied the hotspot detection using a ML based tool called “you only look once” (YOLO). I–V measurements and IRT images (using the Fluke TiS 10 IRT camera with an external DC power supply) of 15 PV modules of 3 different companies (5 PV modules per company) were used for the analysis. The data and images were collected under laboratory condition. Furthermore, two subsets were used to evaluate the results, with the first using 10 IRT images and the second 14 images. Their findings demonstrated that the detector could detect hotspots meticulously as the dataset could be improved and the time it takes the detection could make possible real-time detection. Additional versions of the YOLO were also used to minimize the localization errors (i.e., YOLOv2 and YOLOv3). The developed detector was able to detect most of the hotspots in a given module meticulously. However, even if the training datasets used were generally small, it was observed that the addition of several more IRT images could significantly increase the detection accuracy of the detector.

Di Tommaso, A. et al. in Ref. [37] proposed a multi-stage model based on YOLOv3 network and computer vision techniques for fault diagnosis of PV modules using IRT and visible imaging captured by a UAV. The proposed model could a) detect the location of a defect, b) classify different defect types (i.e., hotspots, bird dropping, raised rooftop panels, delamination, soiling, strong soiling, and stagnant water), and c) predict the severity of hotspot areas (temperature gradients), and the soiling coverage occurring to a PV module (visual images). By using such information, the model was also capable of suggesting maintenance actions. The UAV used consisted of a Sigma Ingegneria Efest MKII drone with a DJA A2 flight controller, Workswell WIRIS 640 s Gen (thermal IRT camera), and MAPIR Survey3N RGB (visible RGB camera). Inspections were performed to two PV plants located in Sicily and Campania in the south Italy, of 9 MW_p_ (2038 captured images) and 21 MW_p_ (1500 captured images) respectively. The datasets were randomly split into 70:15:15 % for the train, validation, and test set, respectively. The accuracy metrics in this study were presented as function of IoU (e.g., average precision (AP) at 0.50 IoU, AP@0.50 equal to 80 %). An AP@0.50 exceeding 98 % was achieved for panel detection. The model yielded a AP@0.40 (AP@0.50) of roughly 88.30 % (66.90 %) for hotspots and a mAP@0.50 of almost 70 % for soiling, bird dropping, delamination, presence of puddles and raised rooftop panel.

Lastly, in the literature the need for fully functional diagnostic tools, that are cable to accurately detect and classify failure modes in PV systems and improve O&M, is evident. However, different challenges arise especially when it comes to large-scale PV power plants, such as the developed measurement strategies for high-quality data, and the relevance of the detected anomalies on decision making, which is of vital importance for PV stakeholders [9]. A solution to these challenges, that can improve the decision making for the necessary O&M, is the development of standards, elaborated scientific studies and most important development of available suitable datasets for adapting, training and test of existing computational tools for PV applications [9].

## 5 Results and discussion

In this review paper, an overview of different failure modes affecting PV plants as well as diagnostic techniques used throughout literature were provided. In particular, the main reported techniques were visual inspection, image techniques and analysis of the recorded PV electrical data. Each category has its merits and drawbacks. Image techniques and electrical data analytic methods gained more attention in the past years, as these techniques can automate the PV inspection and diagnosis procedure, thus reducing time and cost. Another important milestone for PV plant's inspection was the introduction of UAV systems. UAV systems present unique advantages and opportunities in the field of fault detection of PV systems due to the large area coverage, high flexibility, and fast speed for inspection.

Table 6 summarizes the procedures (i.e., data acquisition method) and techniques (visual inspection, image techniques and electrical data analysis) used in literature for fault diagnosis.

**Table 6:** Overview of fault diagnosis (visual inspection, IRT, RGB and EL imagery, and data analytics) and data acquisition methods (manual: indoor or/and outdoor acquisition, and automatic: performance data or/and UAV outdoor acquisition) reported in the literature.

REF.	FAULT DIAGNOSIS METHOD					DATA ACQUISITION METHOD			
	Visual Inspection	Imaging			Electrical data analysis	Manual		Automatic	
		IRT	RGB	EL		Indoor	Outdoor	Performance data	UAV outdoor
[3]					✓		✓		
[4]									
[7]					✓			✓	
[11]		✓			✓		✓	✓	
[14]		✓		✓	✓	✓			
[16]					✓			✓	
[17]					✓			✓	
[18]					✓			✓	
[19]					✓			✓	
[20]					✓			✓	
[22]				✓			✓		✓
[23]		✓							✓
[25]			✓				✓		
[26]				✓		✓			
[27]		✓							✓
[29]		✓							✓
[31]					✓			✓	
[32]					✓			✓	
[33]					✓			✓	
[34]		✓	✓						✓
[35]					✓			✓	
[36]					✓			✓	
[37]		✓	✓						✓
[38]					✓			✓	
[43]					✓			✓	
[44]		✓				✓			
[47]	✓	✓			✓	✓	✓		
[51]					✓			✓	
[53]		✓					✓		
[54]					✓			✓	
[58]		✓	✓						✓
[59]		✓					✓		
[61]		✓							✓
[62]		✓	✓						✓
[64]		✓							✓
[67]		✓					✓		
[69]		✓							✓
[70]		✓							✓
[71]		✓					✓		✓
[72]		✓	✓						✓
[73]			✓		✓		✓		
[74]		✓	✓						✓
[75]		✓	✓						✓
[76]		✓	✓						✓
[77]			✓						✓
[78]			✓						✓
[79]			✓						✓
[80]			✓						✓
[81]			✓				✓		
[82]		✓	✓				✓		✓
[83]				✓		✓			
[84]				✓					✓
[85]					✓			✓	
[86]				✓		✓			
[87]				✓		✓	✓		
[88]				✓			✓		
[89]				✓		✓			
[91]		✓					✓		✓
[93]					✓			✓	
[95]					✓			✓	
[97]					✓			✓	
[98]					✓			✓	
[99]					✓			✓	
[100]					✓			✓	
[110]		✓	✓	✓	✓	✓	✓		✓
[111]		✓					✓		
[112]		✓							✓
[113]			✓						✓
[114]		✓	✓						✓
[130]		✓	✓						✓
[131]		✓	✓				✓		✓
[141]		✓							✓
[139]			✓						✓
[142]		✓					✓		✓
[143]		✓	✓						✓
[144]					✓			✓	
[145]					✓			✓	
[146]					✓			✓	
[148]		✓							✓
[149]					✓			✓	
[151]					✓			✓	
[106]		✓					✓		
[107]		✓					✓		
[108]		✓							✓
[152]		✓							✓
[153]		✓				✓			
[154]			✓						✓
[156]				✓			✓		
[105]				✓		✓			
[158]				✓		✓			
[159]				✓					✓
[160]		✓	✓		✓				✓
[162]		✓			✓	✓			
[163]				✓			✓		
[164]	✓	✓			✓		✓	✓	
[165]	✓	✓		✓	✓	✓	✓	✓	
[166]		✓							✓
[167]		✓							✓
[168]		✓							✓
[169]				✓					✓
[170]		✓							✓
[171]		✓							✓
[172]		✓							✓
[173]					✓			✓	
[174]		✓	✓				✓		✓
[175]		✓	✓						✓
[176]		✓	✓						✓
[177]		✓							✓

**Fig. 11:**
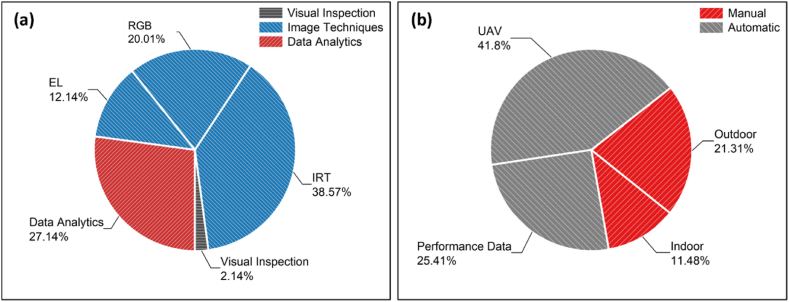
Overview of the 108 investigated studies for fault diagnosis procedures in PV systems using different (a) fault diagnosis methods (visual inspection, image techniques using: IRT, RGB and EL images, and electrical data analytics), and (b) data acquisition used (manual: indoor or/and outdoor acquisition, and automatic: performance data or/and UAV acquisition).

It can be observed that 70.72 % of the reported diagnostic algorithms are based on image analysis techniques (see Fig. 11a). In addition, IRT is the most used imaging analysis technique (38.57 %), followed by the analysis of RGB (20.01 %) and EL (12.14 %) images. Moreover, the preference to automatic (e.g., UAV and performance data) over manual (outdoor and indoor) data acquisition method is evident throughout the literature (see Fig. 11b). Automatic data acquisition methods were used in 67.21 % of the investigated studies.

**Fig. 12:**
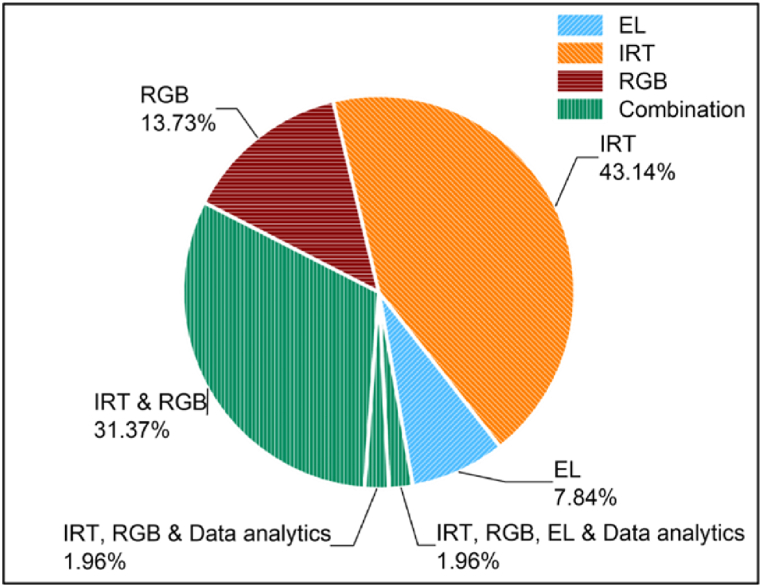
Overview of the 51 investigated studies which used UAV for the acquisition of data for fault diagnosis in PV systems. Fault diagnosis methods used: EL, IRT, RGB images and combination of methods.

An overview of the different fault diagnosis methods used in combination with UAV acquisition is illustrated in Fig. 12. RGB images are usually used in combination with IRT (31.37 %). This is because a significant range of failure modes can be identified by employing both of these methods together. Also, IRT and RGB imagery are the most straightforward to be used on a fielded operating PV system rather than the EL, especially when using UAV means. Few studies used EL images (7.84 %) acquired by a UAV, showing that it is feasible but different problems encountered due to the nature of the EL procedure. Finally, electrical data analytic methods are widely used for PV plant diagnostics which can be found as a stand-alone technique (27.14 % as depicted in Fig. 11) or complemented by image analysis techniques. A considerable number of studies (approximately 35 %) including UAV acquisition were using a combination of methods for fault diagnosis of PV plants. Finally, only a handful of studies (∼2 %) were found using all the imaging techniques and data analytics in combination with UAV acquisition.

## 6 Conclusions

Accurate fault identification is critical for reducing investment risk and increasing the PV technology's bankability. The implementation of fault diagnostic tools on operating systems is nowadays necessary to ensure optimal PV performance. The fault diagnostic results can be then processed to organise/optimise the field activities and to take the appropriate corrective actions for resolving the fault occurrences, thus minimising power losses and maximising the energy production.

This study provides insights into the existing approaches for PV plant diagnosis. 10.13039/501100014065Focus was shed on UAV-based approaches, that can support PV plant diagnostics using imaging techniques and data analytics. In this context, the essential equipment needed and the sensor requirements (parameters and resolution) for the diagnosis of failures in monitored PV systems using UAV-based approaches were outlined. Moreover, this review revealed the different types of failures that can be detected by such approaches, and it provided recommendations on how to develop/construct a fully functional UAV-based diagnostic tool, capable of diagnosing accurately failure modes in PV systems.

The extensive review demonstrated that image techniques are the most common fault diagnostic analysis method. Furthermore, IRT is the preferable imaging technique (38.57 %), followed by the analysis of RGB (20.01 %) and EL (12.14 %) images. Approximately 67 % of the reported studies make use of automatic data acquisition methods (i.e., 41.80 % UAV acquisition and 25.41 % performance data acquisition). Furthermore, IRT also prevails as the main technique used in combination with UAV acquisition, with a 43.14 % as a stand-alone technique and 35.29 % in combination with other techniques. On the other hand, EL is the least used imaging technique (12.14 %), with only few studies (7.84 %) using EL images acquired by a UAV which is attributed to the nature of the EL procedure. Another widely used technique for PV plant diagnostics is data analytics which can be found as a stand-alone technique (27.14 %) or as a complementing technique to imaging methods (∼4 % of the investigated studies using UAV acquisition). Lastly, it is evident that the use of all the imaging techniques and data analytic methods in combination with UAV acquisition is not fully embraced in the research community with only few studies (∼2 %) found in literature.

Concluding, the use of UAV diagnostic systems can improve the overall O&M procedures and reduce cost (O&M and LCOE) by increasing the inspection efficiency up to 97 % on average compared to the manual inspection [26]. Furthermore, autonomous flight strategies were found to further reduce the operation time of a UAV by approximately 60 % [64]. The development of accurate fault diagnosis procedures could avoid economic losses up to 7.4 million $/year for an 1 GW_p_ PV plant (with approximately 4 % losses in energy yield) [30]. Therefore, fault diagnosis based on combination of techniques and UAV acquisition could provide early diagnosis on multiple fault defects assuring improved PV performance and preventing defects’ extension to healthy areas.

## Data availability

No data was used for the research described in the article.

## CRediT authorship contribution statement

**Anna Michail:** Writing – review & editing, Writing – original draft, Visualization, Validation, Software, Methodology, Investigation, Formal analysis, Conceptualization. **Andreas Livera:** Writing – review & editing, Writing – original draft, Validation, Methodology, Investigation, Formal analysis, Conceptualization. **Georgios Tziolis:** Writing – review & editing, Writing – original draft, Methodology, Investigation, Conceptualization. **Juan Luis Carús Candás:** Writing – review & editing, Writing – original draft, Visualization, Validation, Resources, Project administration, Methodology, Investigation, Funding acquisition, Formal analysis, Conceptualization. **Alberto Fernandez:** Writing – review & editing, Writing – original draft, Methodology, Investigation. **Elena Antuña Yudego:** Writing – review & editing, Writing – original draft, Methodology, Investigation. **Diego Fernández Martínez:** Writing – review & editing, Writing – original draft, Resources, Formal analysis. **Angelos Antonopoulos:** Writing – review & editing, Writing – original draft, Methodology, Formal analysis. **Achilleas Tripolitsiotis:** Writing – review & editing, Writing – original draft, Methodology, Investigation, Formal analysis. **Panagiotis Partsinevelos:** Writing – review & editing, Writing – original draft, Methodology, Investigation. **Eftichis Koutroulis:** Writing – review & editing, Writing – original draft, Supervision, Resources, Project administration, Methodology, Investigation. **George E. Georghiou:** Writing – review & editing, Writing – original draft, Supervision, Resources, Project administration.

## Declaration of competing interest

The authors declare that they have no known competing financial interests or personal relationships that could have appeared to influence the work reported in this paper.
